# Multicomponent intranasal adjuvant for mucosal and durable systemic SARS-CoV-2 immunity in young and aged mice

**DOI:** 10.1038/s41541-023-00691-1

**Published:** 2023-06-29

**Authors:** Sonia Jangra, Jeffrey J. Landers, Gabriel Laghlali, Raveen Rathnasinghe, Prajakta Warang, Seok-Chan Park, Jessica. J. O’Konek, Gagandeep Singh, Katarzyna W. Janczak, Adolfo García-Sastre, Nandini Arya, Dilara Karadag, James R. Baker, Michael Schotsaert, Pamela T. Wong

**Affiliations:** 1 Department of Microbiology, Icahn School of Medicine at Mount Sinai, New York, NY USA; 2 Global Health and Emerging Pathogens Institute, Icahn School of Medicine at Mount Sinai, New York, NY USA; 3 Department of Internal Medicine, University of Michigan Medical School, Ann Arbor, MI USA; 4 Michigan Nanotechnology Institute for Medicine and Biological Sciences, University of Michigan Medical School, Ann Arbor, MI USA; 5 Mary H. Weiser Food Allergy Center, University of Michigan Medical School, Ann Arbor, MI USA; 6 Graduate School of Biomedical Sciences, Icahn School of Medicine at Mount Sinai, New York, NY USA; 7 Laboratory of Pathology, College of Veterinary Medicine, Jeonbuk National University, Iksan, 54596 Korea; 8 Biosafety Research Institute, College of Veterinary Medicine, Jeonbuk National University, Iksan, 54596 Korea; 9 Department of Medicine, Division of Infectious Diseases, Icahn School of Medicine at Mount Sinai, New York, NY USA; 10 The Tisch Cancer Institute, Icahn School of Medicine at Mount Sinai, New York, NY USA; 11 Department of Pathology, Molecular and Cell-Based Medicine, Icahn School of Medicine at Mount Sinai, New York, NY USA

**Keywords:** Adjuvants, Viral infection, Pattern recognition receptors

## Abstract

Multiple FDA-approved SARS-CoV-2 vaccines currently provide excellent protection against severe disease. Despite this, immunity can wane relatively fast, particularly in the elderly and novel viral variants capable of evading infection- and vaccination-induced immunity continue to emerge. Intranasal (IN) vaccination more effectively induces mucosal immune responses than parenteral vaccines, which would improve protection and reduce viral transmission. Here, we developed a rationally designed IN adjuvant consisting of a combined nanoemulsion (NE)-based adjuvant and an RNA-based RIG-I agonist (IVT DI) to drive more robust, broadly protective antibody and T cell responses. We previously demonstrated this combination adjuvant (NE/IVT) potently induces protective immunity through synergistic activation of an array of innate receptors. We now demonstrate that NE/IVT with the SARS-CoV-2 receptor binding domain (RBD), induces robust and durable humoral, mucosal, and cellular immune responses of equivalent magnitude and quality in young and aged mice. This contrasted with the MF59-like intramuscular adjuvant, Addavax, which showed a decrease in immunogenicity with age. Robust antigen-specific IFN-γ/IL-2/TNF-α was induced in both young and aged NE/IVT-immunized animals, which is significant as their reduced production is associated with suboptimal protective immunity in the elderly. These findings highlight the potential of adjuvanted mucosal vaccines for improving protection against COVID-19.

## Introduction

The COVID-19 pandemic caused by severe acute respiratory syndrome coronavirus-2 (SARS-CoV-2) continues to exact a severe toll on global human health three years since initial identification of the virus. Several highly effective vaccines have been deployed which effectively reduced the morbidity and mortality of COVID-19^1^. However, the continued emergence of highly mutated and more transmissible SARS-CoV-2 variants of concern (VOCs) capable of evading vaccine- and infection-based immunity has posed an ongoing threat, especially for vulnerable groups including the immune compromised and the elderly^2^. These concerns were underscored by the rapid surge in infections in both naïve and immunized populations upon emergence of the Omicron lineage^3,4^. New VOCs continue to emerge, which will further challenge current levels of immunity. Moreover, evidence demonstrating waning of immunity imparted by current mRNA and adenovirus vectored vaccines, particularly in elderly populations, highlights the need for vaccine strategies which impart broad and long-lasting immunity in both young and aged populations^5–8^.

There has been much interest in developing mucosal vaccines which induce both systemic and respiratory mucosal immunity against SARS-CoV-2. This is especially pertinent given the significant levels of breakthrough infections and viral transmission observed within vaccinated populations. Recent studies indicated that while vaccinated populations generally clear virus faster, at early time points post-infection, significant levels of infectious virus could be found in the nasopharynx of both vaccinated and unvaccinated individuals^9^. Even if complete sterilizing immunity is not achieved, mucosal immunity in the upper respiratory tract provides the major advantage of blocking viral dissemination into the lower respiratory tract, and memory T cells within the respiratory tract can provide more effective protection^10–14^.

Here we expand on our previous work on a rationally designed combination intranasal (IN) adjuvant composed of a nanoemulsion-based adjuvant (NE) and an RNA-based agonist of RIG-I (IVT DI)^15,16^. NE is an oil-in-water emulsion consisting of soybean oil, a nonionic (Tween80) and cationic (cetylpyridinium chloride) surfactant, and ethanol^17,18^. This adjuvant has established Phase I clinical safety as an IN adjuvant in two different human trials^19–21^. NE induction of mucosal and systemic immune responses is mediated, at least in part, through TLR2 and 4 activation and through NLRP3 activation via induction of immunogenic apoptosis^22,23^. IVT DI is a highly selective RIG-I agonist, derived from the full-length (546nt) copy-back defective interfering (DI) RNA of Sendai virus (SeV) strain Cantell^24,25^. Together, the combined adjuvant (NE/IVT) thus contains agonists for all three innate receptor classes (TLRs, RLRs, NLRs) necessary for the induction of potent antiviral immune responses. As activation of not only a robust, but also tailored innate immune response is critical for shaping an effective and durable adaptive response, such targeted adjuvants are expected to drive improved immune responses particularly in the context of immunosenescence.

We have previously demonstrated that NE/IVT induces marked synergistic activation of innate immune responses both through co-activation of these three receptor classes and through the facilitation of IVT cellular uptake through the RNA carrier properties of NE^16^. IN administration of NE/IVT activates synergistic innate and adaptive immune responses through these pathways, resulting in improved protective immunity against influenza virus and SARS-CoV-2 when using an inactivated virus or recombinant subunit (S1 subunit of the spike (S) protein) antigens, respectively^15,16^. We now examine the impact of aging on the immune responses induced by NE/IVT, by evaluating the ability of NE/IVT to induce broad protective immune responses to SARS-CoV-2 using a less immunogenic recombinant antigen, the receptor binding domain (RBD) of the S protein. We demonstrate that NE/IVT induces broad and robust serum-neutralizing antibody (nAb) responses against multiple VOCs along with strongly T_H_1 polarized T cell recall responses and mucosal immune responses independent of aging. Induced humoral immune responses were protective against mouse adapted and antigenically drifted SARS-CoV-2 viruses after passive serum transfer. This contrasted with what was observed with the MF59-like adjuvant Addavax, a squalene-based intramuscular (IM) oil-in-water adjuvant. Addavax immunized mice showed a significant decline in humoral and cellular immune responses in aged animals and did not induce measurable mucosal responses. Moreover, IN NE/IVT provided enhanced protection from morbidity in vaccinated, senescent animals upon heterologous challenge as compared to IM Addavax. Finally, we demonstrate that the humoral and cellular immune responses induced by NE/IVT are both durable and long-lived. These findings are significant, as MF59 is the licensed adjuvant currently used in influenza vaccines for the elderly and highlight the potential of the mucosal NE/IVT adjuvant for improving vaccine responses in this population.

## Results

### Intranasal immunization with NE and NE/IVT induces robust humoral immune responses and elicits mucosal antibody responses in both young and aged mice

The ability of NE and NE/IVT to induce immune responses in the context of aging was examined in young (2 months old (m.o.) at initiation, 4.5 m.o. at completion) and aged (8 m.o. at initiation, 10.5 m.o. at completion) mice. Recombinant, monomeric SARS-CoV-2 spike protein receptor binding domain (RBD) was selected as antigen to better differentiate responses, as it has lower immunogenicity than either the full-length spike (S) protein or the S1 subunit previously examined with the NE/IVT adjuvant^15^. The RBD contains the region of the S protein necessary for binding to the human ACE2 receptor (hACE2) and for viral entry, and thus contains most epitopes targeted by neutralizing antibodies (nAbs) as well as multiple T cell epitopes^26–28^.

**Fig. 1:**
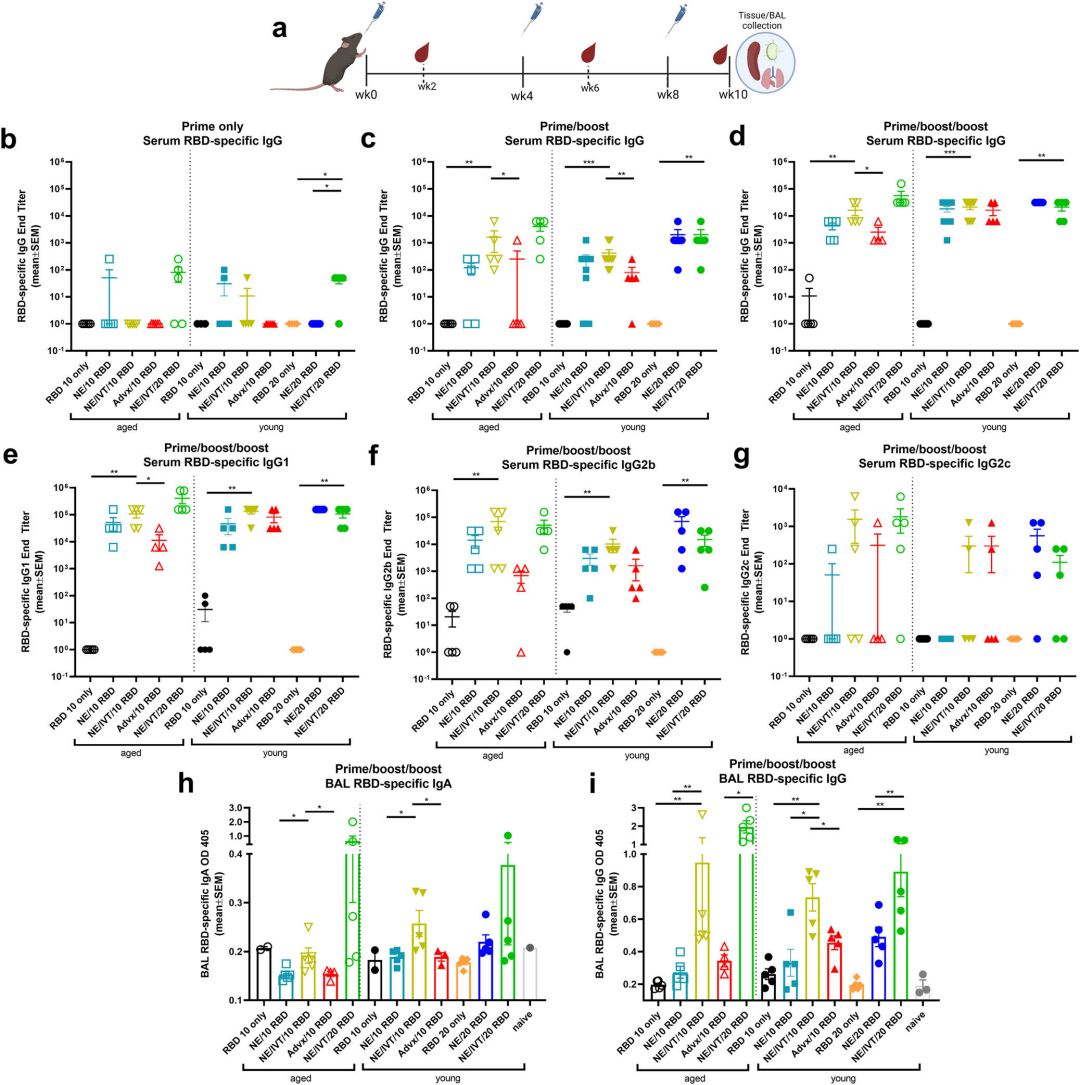
RBD-specific humoral and mucosal immune responses induced in aged versus young mice. Young (8wk) and aged (8mo) mice were immunized IN with 10 or 20 μg RBD with PBS, NE, or NE/IVT, or IM with RBD with Addavax. **a** Study design including vaccination and sampling time points. Mice were given three immunizations at a 4-week interval. RBD-specific IgG titers were measured in sera by ELISA two weeks after (**b**) prime (**c**) prime/boost and **d** prime/boost/boost immunizations. Serum RBD-specific IgG subclass titers for (**e**) IgG1, **f** IgG2b, **g** IgG2c, were measured after the last immunization. Titers are shown as mean ± SEM (*n* = 5/grp) (**h**) Mucosal RBD-specific IgA and **i** IgG were measured in BAL at week 10. (*p < 0.05, **p < 0.01 by Mann–Whitney *U* test shown only for NE/IVT/10 RBD compared to other treatments in the same age group, and for NE/IVT/20 RBD compared only to high dose RBD groups within the young group. Full statistical analysis is shown in Supplementary Table 1).

Mice were given three IN immunizations of RBD with either PBS, 20% NE, or 20% NE/0.5 μg IVT in a total volume of 12 μL, such that the administered vaccine remained within the nasal cavity. RBD was tested in two doses: 10 μg in both young and aged mice for all adjuvant groups, and 20 μg in select groups (with NE and NE/IVT in young mice or with NE/IVT in aged mice) to examine dose effect (RBD 10 only, RBD 20 only, NE/10 RBD, NE/20 RBD, NE/IVT/10 RBD, NE/IVT/20 RBD). Immunizations were performed according to a prime/boost/boost schedule with a four-week (wk) interval between each administration as shown in Fig. 1a. Mice immunized intramuscularly (IM) with 10 μg RBD and 50% Addavax, an MF59-like adjuvant, were included for comparison (IM Advx/10 RBD). Serum RBD-specific IgG titers were measured two weeks after each immunization at weeks 2, 6, and 10 (Fig. 1b–d). Minimal antigen-specific IgG was detected in aged mice after one immunization. No RBD-specific IgG was detectable in aged treatment groups given RBD 10 only, NE/IVT/10 RBD, or IM Advx/10 RBD. Aged groups given the higher dose RBD with the combined NE/IVT adjuvant (NE/IVT/20 RBD) were found to have low but detectable IgG titers (≤1:250) in 3 out of 5 mice, and the NE/10 RBD group induced detectable titers in only 1 out of 5 mice (Fig. 1b). Similarly, in young mice, no RBD-specific IgG was detectable for groups given RBD 10 only or IM Advx/10 RBD. However, low but detectable RBD-specific IgG was detectable in some of the young mice in the NE/10 RBD (2/5), and NE/IVT/10 RBD (1/5) treatment groups at this early time point. For animals given the higher dose of RBD, no RBD-specific IgG was detected in the RBD alone, or NE/20 RBD groups, whereas low but significant IgG titers were induced in most mice in the combined adjuvant NE/IVT/20 RBD group (4/5), demonstrating the advantage of the combined NE/IVT adjuvant in both young and aged animals.

After the second immunization (prime/boost), IgG titers increased in all adjuvanted groups in both age cohorts (Fig. 1c). Titers were similar in young and aged mice given NE/10 RBD (GMT 43, 23, respectively) and in young and aged mice given NE/IVT/10 RBD (GMT 546, 287, respectively), with slightly higher titers induced by the combined adjuvant as compared to the single NE adjuvant in both age cohorts. Significantly lower titers were observed in both age cohorts for IM Advx/10 RBD groups compared to the IN NE and NE/IVT adjuvanted groups at the same antigen dose. Importantly, while NE and NE/IVT induced comparable magnitudes of humoral immune responses in young and aged mice, a clear reduction in induced IgG was observed for IM Advx/10 RBD in aged mice, giving undetectable IgG in the majority of mice (4/5) even after two immunizations. NE and NE/IVT with the 20 μg RBD dose induced higher titers of RBD-specific IgG (GMT 1 × 10^3^, 2.4 × 10^3^, respectively) than the corresponding 10 μg RBD groups in both young and aged mice. Importantly, NE/IVT induced comparable, if not higher IgG in the aged mice compared to the young mice. Similar titers also were observed between the NE and NE/IVT IN adjuvants at the higher RBD dose. These results suggest that these two IN adjuvant approaches are superior in inducing strong humoral immune responses that are maintained in the context of aging. While IgG titers for each of the adjuvanted groups were higher by 1–2 logs when measured against the full-length S protein as compared to their RBD-specific titers, the relative pattern in IgG titers between treatment groups was identical (Supplementary Fig. 1). After the third immunization (prime/boost/boost), RBD-specific IgG titers further increased for all adjuvanted groups in young and aged mice (Fig. 1d). Similar high IgG titers were induced in young mice given NE/10 RBD, NE/IVT/10 RBD, and IM Advx/10 RBD (1.2 × 10^4^, 1.6 × 10^4^, 1.2 × 10^4^, respectively). While comparable titers were induced after the final boost in young and aged mice given NE/IVT/10 RBD, titers for aged mice immunized with NE/10 RBD and IM Advx/10 RBD did not increase as much as those for the corresponding young groups, supporting the advantage of the combined NE/IVT adjuvant. In young mice given 20 μg RBD, similar RBD-specific IgG titers were observed for the NE and NE/IVT groups, which were comparable to those in young mice immunized with 10 μg RBD with the same adjuvants. However, for the NE only groups, the spread in induced IgG titers was reduced at the higher RBD dose, suggesting a more optimal response with the higher antigen dose for the single adjuvant. As was observed after the second immunization, similar or higher titers were seen in the aged mice given NE/IVT/20 RBD as compared to the young animals after the third immunization. While no difference in RBD-specific IgG was observed in young mice when comparing the NE/IVT adjuvanted 10 and 20 μg RBD groups, a half-log increase was observed in aged mice with the higher antigen dose.

RBD-specific IgG subclass distributions for IgG1, IgG2b, and IgG2c were analyzed at the 10-week (wk) time point (Fig. 1e–g). Subclass analysis indicated a balanced T_H_1/T_H_2 profile for the NE and NE/IVT adjuvants, consistent with previous studies^15,16,29,30^. IgG1 titers followed the same relative pattern in each treatment group across age cohorts as observed for total IgG. NE/IVT induced higher IgG1 titers than NE alone in both young and aged mice, and both adjuvants induced similar IgG1 titers in young and aged groups (Fig. 1e). Robust IgG2b titers were also induced by the NE and NE/IVT in young and aged mice. As in previous studies, inclusion of IVT in the NE/IVT enhanced IgG2b and 2c titers relative to NE alone, consistent with the greater T_H_1-bias of NE/IVT. Interestingly, NE/10 RBD and NE/IVT/10 RBD induced higher IgG2b titers (half log higher) in aged as compared to young mice. In contrast to the IN adjuvants, IM Advx/10 RBD induced notably less IgG2b relative to IgG1, particularly in aged mice, consistent with the more T_H_2-polarizing properties of this adjuvant. IgG2c titers induced by NE and NE/IVT were lower than IgG2b, appearing to require a higher antigen dose for optimal induction. However, NE/IVT also appeared to induce higher IgG2c in aged mice as compared to young, as particularly evident at the higher RBD dose (NE/IVT/20 RBD) (Fig. 1g).

A major advantage of intranasal vaccines is the induction of mucosal immune responses. To assess mucosal antibodies induced by immunization, bronchoalveolar lavage (BAL) fluid was collected from immunized animals at week 10, and RBD-specific IgA and IgG were measured (Fig. 1h, i). At the 10 μg RBD dose, no antigen-specific IgA was induced in either age cohort immunized IM with Advx/10 RBD, consistent with the poor induction of mucosal responses by parenteral vaccination. While no IgA was detected for the RBD 10 only or NE/10 RBD groups in either age group, the NE/IVT/10 RBD induced significant levels of IgA in young mice. However, this effect was diminished in aged mice. At the high RBD dose, while low levels of IgA were observed in young mice given IN NE/20 RBD, these levels were still lower than those induced by the NE/IVT/10 RBD. The combined adjuvant with the high RBD dose (NE/IVT/20 RBD) induced the highest levels of RBD-specific IgA and induced similar magnitudes of mucosal responses in both young and aged groups. In addition to BAL IgA, IgG in the BAL also contributes to the protection at the mucosa. BAL IgG showed a similar overall pattern as the BAL IgA, with a significant enhancement in RBD-specific IgG observed in the NE/IVT groups compared to the NE alone in both age cohorts. BAL IgG levels remained similar between respective young and aged treatment groups and were elevated in the aged NE/IVT groups compared to the young as was observed for serum IgG. In contrast to IgA, IM Advx/10 RBD also induced detectable levels of BAL IgG, which is consistent with the different sources of production of the IgG and IgA present in the mucosa.

### NE/IVT improves the breadth of neutralizing antibodies against variants of concern in both young and aged mice

**Fig. 2:**
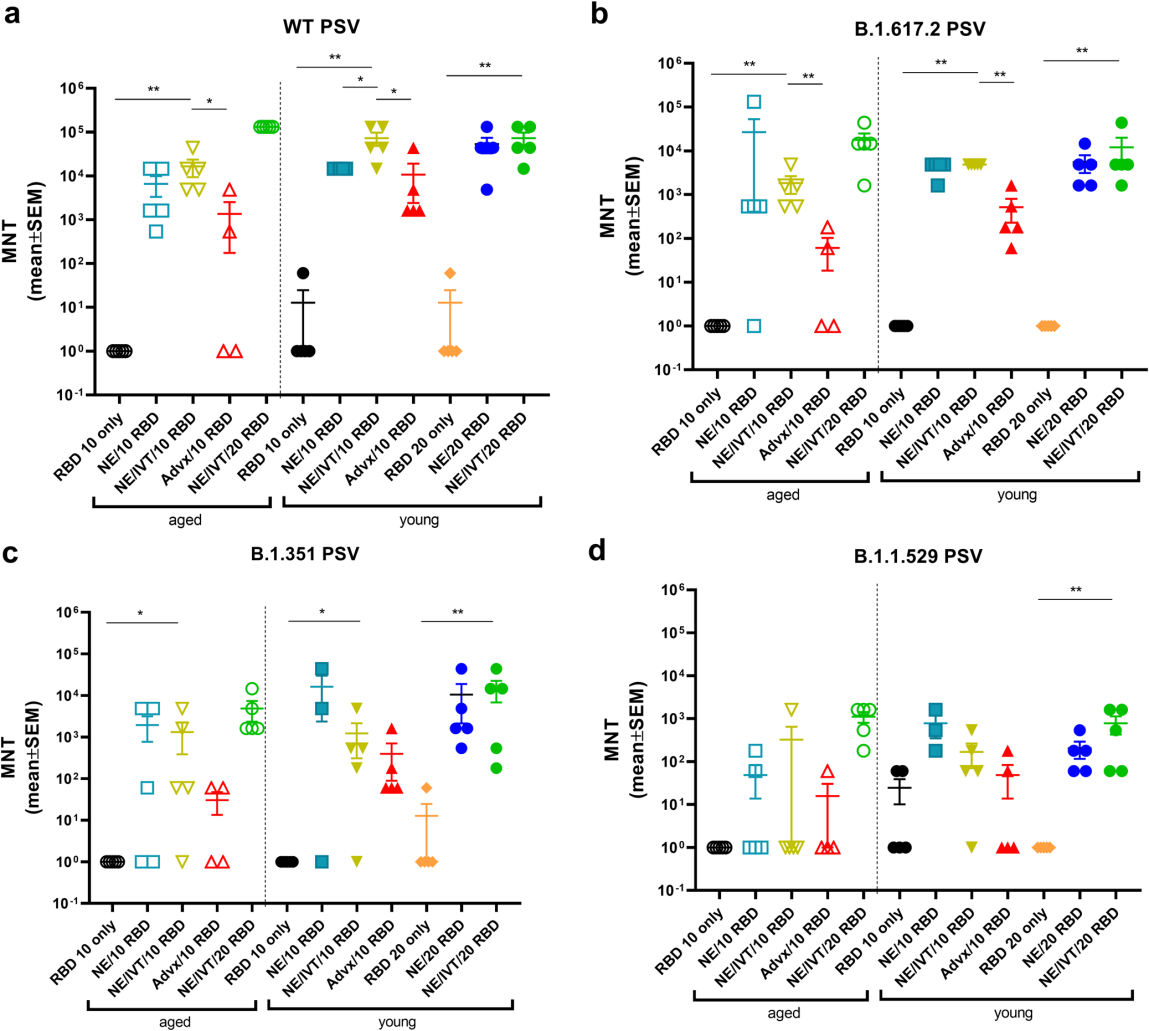
Serum viral neutralizing antibody titers in aged vs. young, immunized mice. Neutralizing antibody titers measured in sera from mice immunized three times IN with 10 or 20 μg RBD with PBS, NE, or NE/IVT, or IM with RBD with Addavax using lentivirus PSVs expressing SARS-CoV-2 S protein derived from (**a**) WT, **b** B.1.617.2, **c** B.1.351, **d** B.1.1.529 viral variants. Titers are shown as mean ± SEM (*n* = 5/grp) (*p < 0.05, **p < 0.01 by Mann–Whitney *U* test shown only for NE/IVT/10 RBD compared to other treatments in the same age group, and for NE/IVT/20 RBD compared only to high dose RBD groups within the young group. Full statistical analysis is shown in Supplementary Table 1).

To evaluate the virus neutralization capability of antibodies induced by the NE and NE/IVT in young and aged mice, neutralizing antibody (nAb) titers were measured in the serum from immunized mice two weeks after the third immunization using a lentivirus-based pseudovirus (PSV) neutralization assay (Fig. 2). Sera were incubated with PSVs expressing spike proteins from either the WT, B.1.617.2, B.1.351, or B.1.1.529 (BA.1) variants, and entry into hACE2 expressing HEK293T cells was quantified as a function of transduction of a luciferase reporter gene. At the 10 μg RBD dose, robust nAb titers were induced by NE and NE/IVT in both young and aged mice against the homologous WT PSV, with the combined adjuvant eliciting marginally higher nAb titers than the single adjuvant in both age groups (Fig. 2a). nAb titers against the WT virus induced by NE/10 RBD and NE/IVT/10 RBD were slightly lower in aged mice as compared to the corresponding young groups, however, titers remained robust in both groups (NE/10 RBD GMT 1.5 × 10^4^ (young) 3.1 × 10^3^ (aged); NE/IVT/10 RBD GMT 5.5 × 10^4^ (young) 1.17 × 10^4^ (aged)). IM Advx/10 RBD induced lower nAb titers against the WT virus in young mice than either NE or NE/IVT groups. Notably, IM Advx/10 RBD induced markedly lower (97-fold) WT nAb titers in aged mice than in young mice, with some aged mice showing no detectable neutralization despite having similar RBD-specific IgG titers as aged mice immunized with NE/10 RBD (Fig. 1d). These results suggest the quality of antibodies elicited by NE and NE/IVT is superior to the those elicited by Addavax, particularly in the context of aging. Similarly, while NE/10 RBD and NE/IVT/10 RBD elicited similar RBD-specific IgG titers in young mice, the higher nAb titers observed with NE/IVT suggest improved antibody quality. For the WT virus, in young mice, increasing the RBD dose increased the nAbs induced by the single NE adjuvant to levels equivalent to the combined adjuvant at the low dose (NE/IVT/10 RBD). Titers did not further increase in young mice at the higher RBD dose for NE/IVT/20 RBD relative to NE/IVT/10 RBD. However, in aged mice, NE/IVT/20 RBD induced nAb titers equivalent to those induced in young mice (GMT 5.5 × 10^4^ (young), 1.3 × 10^5^ (aged)), better maintaining neutralizing capacity than the other adjuvant/antigen combinations which showed variable degrees of reduced viral neutralization in the context of aging.

Similar trends in nAbs were observed against B.1.617.2 (Fig. 2b). At the low RBD dose NE and NE/IVT induced robust nAb titers against B.1.617.2, inducing GMTs of 3.9 × 10^3^ and 4.9 × 10^3^, respectively in young mice, and GMTs of 4.6 × 10^2^ and 1.3 × 10^3^, respectively in aged mice. These titers are approximately a log reduction in nAb titers relative to the WT virus. IM Advx/10 RBD induced lower nAbs against B.1.617.2 in young mice (GMT 2.8 × 10^2^, a 14-fold reduction compared to WT) than the NE and NE/IVT adjuvants at the same antigen dose and showed significantly lower nAbs against the B.1.617.2 PSV in aged mice (GMT 1.0 × 10^1^). Multiple studies have observed greater reduction in neutralization of the B.1.351 variant by antibodies from mice and humans immunized against the WT spike protein compared to the B.1.617.2 variant. Indeed, a reduction in nAb titers against B.1.351 was observed for the low RBD NE and NE/IVT groups compared to the WT and B.1.617.2 variants in both young and aged mice (Fig. 2c). However, nAb titers induced in these groups were still significantly higher than the IM Advx/10 RBD group in both age cohorts. While NE and NE/IVT induced similar B.1.351 nAb titers in both young and aged mice at the low antigen dose, titers in the IM Advx/10 RBD group were 19-fold lower in aged mice relative to young. In contrast, at the high RBD dose, NE and NE/IVT induced robust cross-neutralizing antibodies in young mice against B.1.351(GMTs 3.1 × 10^3^, 3.9 × 10^3^, respectively), which was maintained in aged mice with the combined adjuvant.

Finally, serum was evaluated against the B.1.1.529/BA.1 variant which contains 15 mutations in the RBD relative to the WT virus (Fig. 2d). Significant reduction in neutralization was observed in all groups. While detectable nAbs were observed in young NE and NE/IVT groups at the low antigen dose, these titers were low (GMTs <5 × 10^2^), and only two out of five mice showed detectable titers in the Advx group. These titers were further reduced in aged mice for all three treatment groups. In contrast, while low nAbs were elicited against B.1.1.529 by NE/IVT/20 RBD, titers were maintained in aged mice (GMT 3.5 × 10^2^ (young), 8.2 × 10^2^ (aged)). Together, these results support enhanced induction of broadly neutralizing antibodies by the combined adjuvant in both young and aged animals. Further optimization of antigen type (S1 subunit, full-length S protein), antigenic match and dose will likely lead to improved cross-variant responses.

### Passive transfer of antibodies from young and aged NE/IVT immunized mice provides protection from SARS-CoV-2 infection in naive mice

**Fig. 3:**
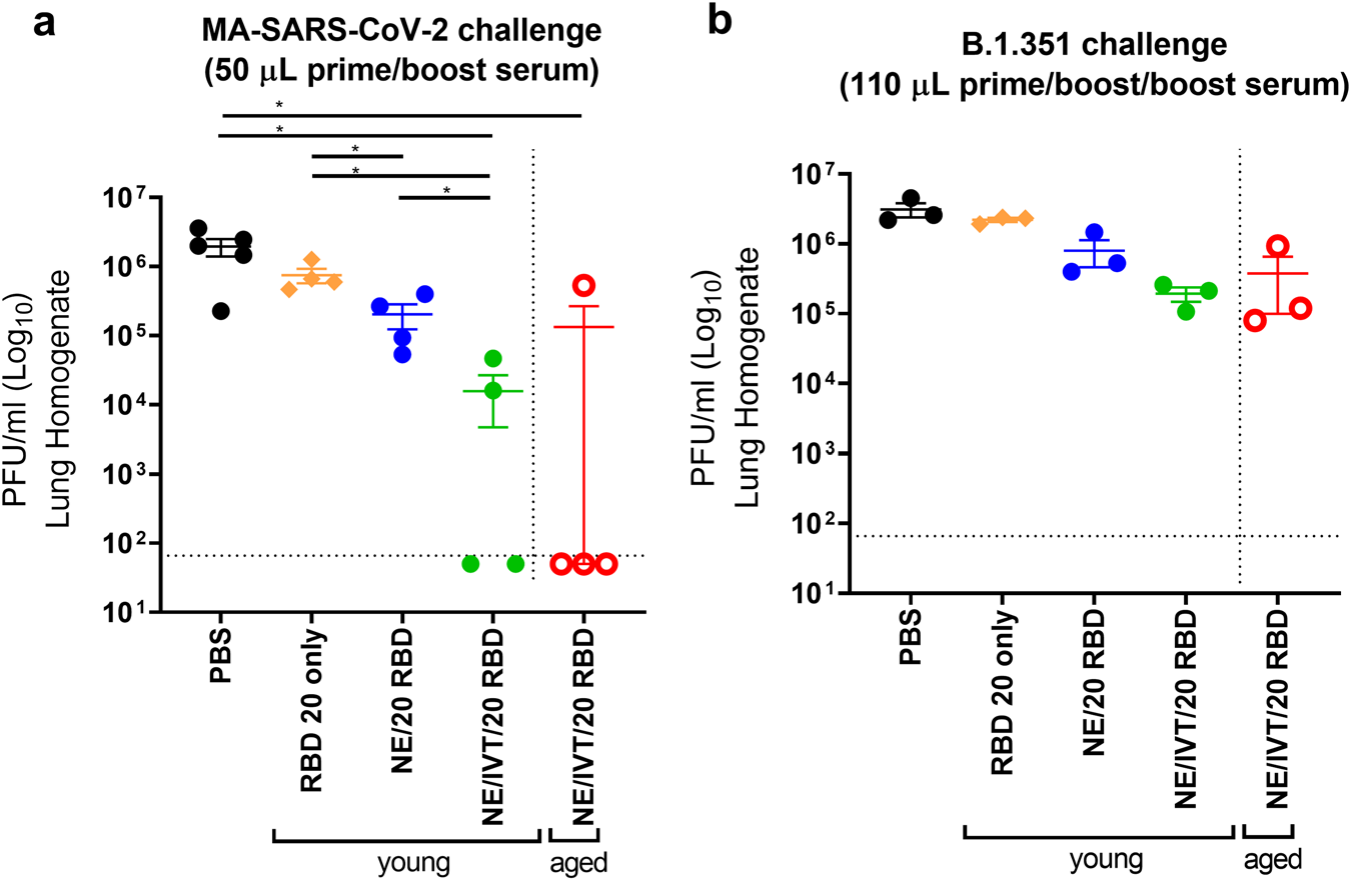
Lung viral titers in naïve mice receiving passive serum transfer from young and aged immunized mice upon challenge with MA-SARS-CoV-2 and B.1.351. **a** Sera from young or aged mice given two IN immunizations with 20 μg RBD with PBS, NE, or NE/IVT were pooled at week 6, and 50 μL of the pooled serum was transferred IP into each naïve mouse 2 h prior to challenge IN with 10^4^ pfu MA-SARS-CoV-2. **b** Sera from young or aged mice given three IN immunizations with 20 μg RBD with PBS, NE, or NE/IVT were pooled at week 10, and 110 μL of the pooled serum was transferred IP into each naïve mouse 2 h prior to challenge IN with 5 × 10^3^ pfu B.1.351. Viral load was assessed in lung homogenate by plaque assay at 3 days post-infection (d.p.i.). The plaque detection limit is indicated by the horizontal dashed line. Lung viral titers are shown as mean ± SEM (*n* = 5/grp) (*p < 0.05, **p < 0.01 by Mann–Whitney *U* test).

To further assess the protection provided by antibodies from young and aged mice immunized with NE and NE/IVT in the absence of cellular immunity, sera from immunized mice were transferred into 8-week old naïve recipient mice. Sera from mice in each immunization group were pooled after two immunizations at week 6 (2 weeks after the last immunization), and 50 μL of pooled serum was transferred intraperitoneally (IP) into each naïve mouse 2 h prior to IN challenge with 10^4^ pfu of mouse-adapted SARS-CoV-2 (MA-SARS-CoV-2) (Fig. 3a and Supplementary Fig. 2A). This low volume of serum was used to maximize observable differences between groups. Viral load was assessed in the lung three days post-infection (dpi). The MA virus was adapted from WT SARS-CoV-2 and contains two amino acid (aa) substitutions in the S protein as compared to the WT virus, including N501Y and H655Y, and a four aa insertion within the S1 subunit^31^. The N501Y substitution, shared amongst the B.1.1.7, B.1.351, P.1 and B.1.1.529 variants, allows MA-SARS-CoV-2 and these variants to use the mACE2 receptor and directly infect WT mice. Minimal reduction in lung viral titer was observed for mice receiving sera from groups immunized with the low RBD dose as compared to the mock treatment group (PBS) after only two immunizations (Supplementary Fig. 2A). Challenged mice receiving sera from young donors immunized IN with NE or NE/IVT, or IM with Advx with 10 μg RBD showed similar degrees of viral load reduction. However, in mice given aged donor serum, NE/IVT/10 RBD appeared to have better reduction in viral titers compared to these other groups even though this reduction was modest (~1 log). At the higher RBD dose, mice receiving serum from young donors immunized IN with NE/20 RBD also showed a reduction of viral titers (Fig. 3a). However, transferred antibodies alone after two immunizations with NE/20 RBD was also insufficient for robust protection against MA-SARS-CoV-2 challenge. In contrast, sera from both young and aged donors immunized IN with NE/IVT/20 RBD provided significant protection in recipient mice, resulting in a marked reduction in viral load in all mice and imparting sterilizing immunity in 2/4 and 3/4 mice, respectively. Thus, while protection remained incomplete after only two immunizations as evaluated with this low serum volume, these results clearly demonstrate that NE/IVT induces higher quality cross-protective antibodies.

We next assessed antibody mediated cross-protection in select groups against B.1.351 which is further divergent from WT than MA-SARS-CoV-2. We evaluated sera from mice given three immunizations (wk10 sera) and transferred a larger volume of pooled sera (110 μL) IP into each naïve recipient 2 h prior to IN challenge with 5 × 10^3^ pfu of B.1.351 (Fig. 3b and Supplementary Fig. 2B). Lung viral load was assessed at 3dpi. Only minimal reduction in lung viral titer was observed as compared to the mock treatment group (PBS) at the low RBD dose with NE, NE/IVT, or Advx with serum from either young or aged donor mice. However, a half-log reduction in viral load was observed in mice receiving serum from the young NE/20 RBD immunized group, and a further 1.5 log reduction in viral load was observed for mice receiving sera from either young or aged mice immunized with NE/IVT/20 RBD. While protection against the divergent B.1.351 variant was not complete, these results clearly suggest a benefit from the combined adjuvant in young and aged mice and are consistent with the relative neutralization titers observed in these immunization groups. Achieving sterilizing immunity will likely be promoted by a combination of both B and T cell responses. The latter may be particularly the case for the RBD antigen, since it has been shown to be less protective as a soluble protein (unconjugated to nanoparticles) than the S1 subunit or full-length S protein, particularly against heterologous viral variants^32^.

### NE/IVT induces strongly T_H_1 polarized antigen recall responses independent of aging

**Fig. 4:**
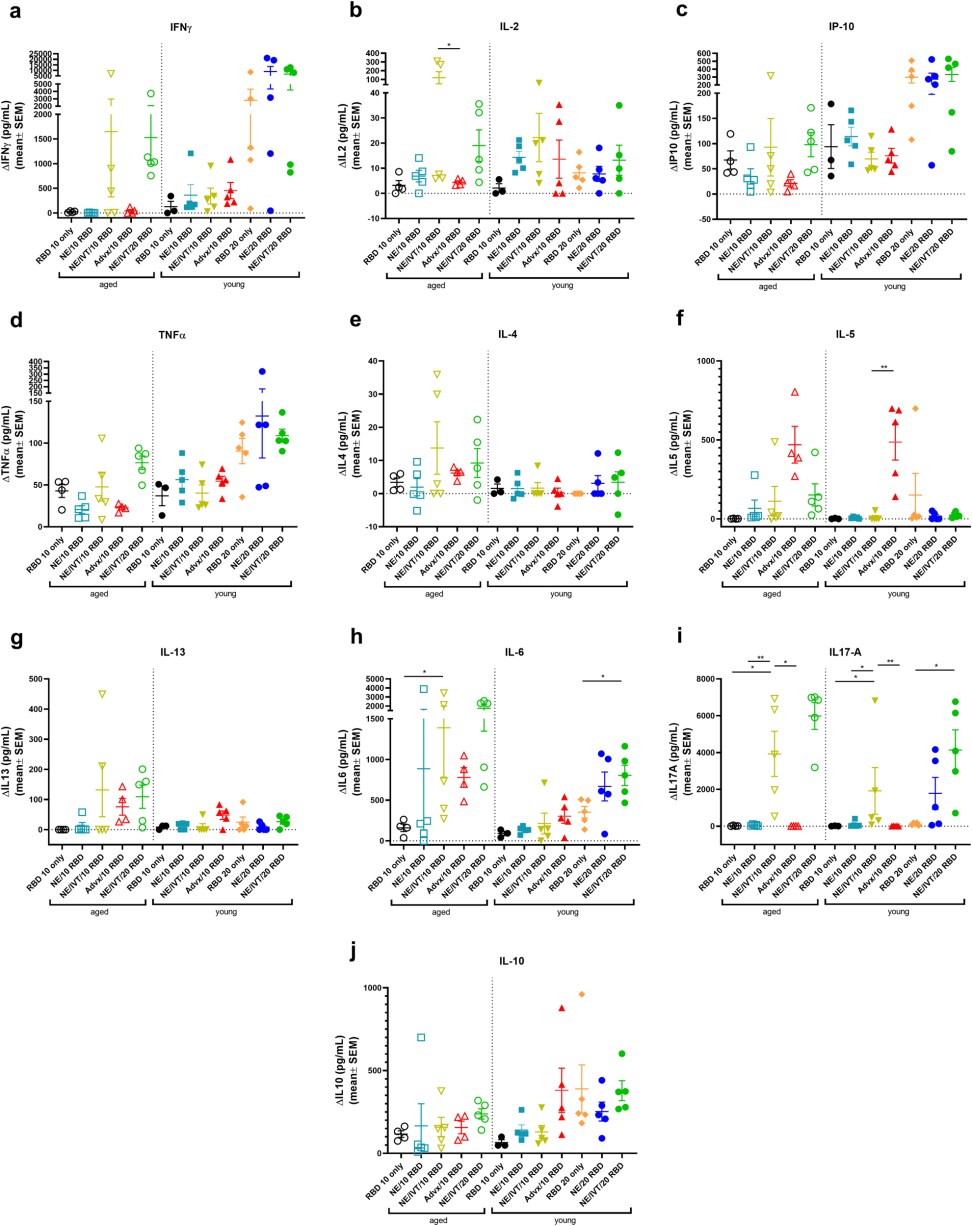
Antigen recall response assessed in splenocytes from aged and young, immunized mice. Splenocytes were isolated from mice given three immunizations with the indicated adjuvant/antigen combinations, two weeks after the final immunization (week 10) and stimulated ex vivo with 5 μg of recombinant RBD for 72 h. Levels of secreted cytokines were measured in the cell supernatant by multiplex immunoassay and compared to unstimulated cells for (**a**) IFN-γ, **b** IL-2, **c** IP-10, **d** TNF-α, **e** IL-4, **f** IL-5, **g** IL-13, **h** IL-6, **i** IL-17A, and **j** IL-10. (data shown as mean ± SEM (*n* = 5/grp)); (*p < 0.05, **p < 0.01 by Mann–Whitney *U* test shown only for select groups for simplicity: NE/IVT/10 RBD compared to other treatments in the same age group, and NE/IVT/20 RBD compared only to high dose RBD groups within the young group. Full statistical analysis is shown in Supplementary Table 1).

**Fig. 5:**
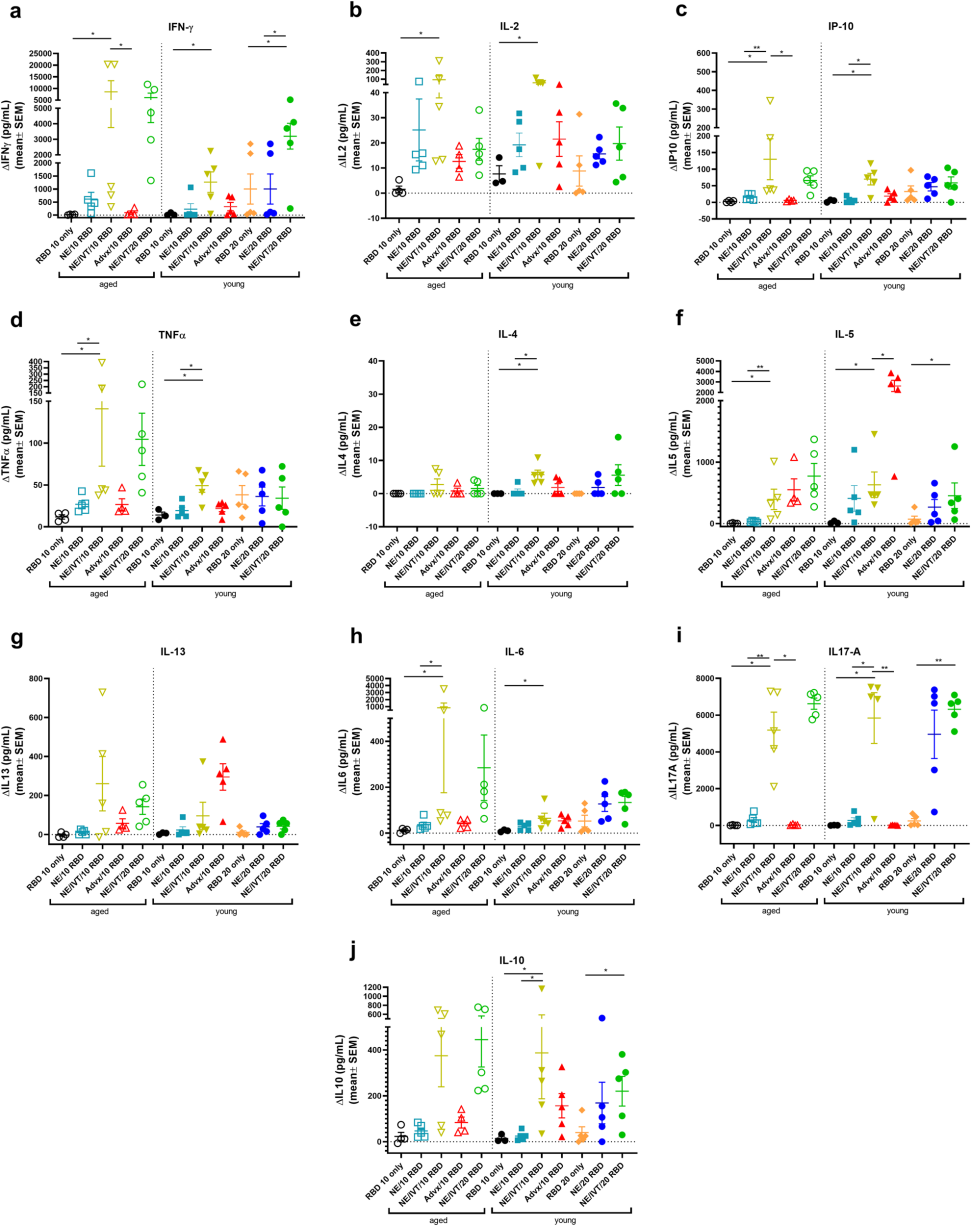
Antigen recall response assessed in cLN from aged and young, immunized mice. cLNs were isolated from mice given three immunizations with the indicated adjuvant/antigen combinations, two weeks after the final immunization (week 10) and stimulated ex vivo with 5 μg of recombinant RBD for 72 h. Levels of secreted cytokines were measured in the cell supernatant by multiplex immunoassay relative to unstimulated cells for (**a**) IFN-γ, **b** IL-2, **c** IP-10, **d** TNF-α, **e** IL-4, **f** IL-5, **g** IL-13, **h** IL-6, **i** IL-17A, and **j** IL-10. (data shown as mean ± SEM (*n* = 5/grp)); (*p < 0.05, **p < 0.01 by Mann–Whitney *U* test shown only for select groups for simplicity: NE/IVT/10 RBD compared to other treatments in the same age group, and NE/IVT/20 RBD compared only to high dose RBD groups within the young group. Full statistical analysis is shown in Supplementary Table 1).

The importance of robust T cell responses in protection against SARS-CoV-2 has been clearly established especially for protection against severe disease. T cell responses are responsible for maintaining immunity when nAbs wane and for imparting immunity against divergent variants that nAbs fail to effectively neutralize given that T cell epitopes are typically more highly conserved across different variants of concern. Accordingly, we evaluated T cell antigen recall responses in the spleen (Fig. 4) and draining lymph nodes (cervical lymph nodes (cLN)) (Fig. 5) of mice immunized with the same RBD/adjuvant formulations as above 2 weeks after the third immunization (week 10). Splenocytes and cells isolated from the cLNs were stimulated ex vivo for 72 h with RBD, and secreted cytokines were measured by multiplex immunoassay and compared to unstimulated cells. Inclusion of IVT DI in the NE/IVT adjuvant dramatically increased the T_H_1 polarization of the cellular response as compared to NE alone, resulting in synergistic enhancement of T_H_1-associated cytokine production in both the spleen and cLN. While IFN-γ production in the spleen for the NE/IVT/10 RBD group was only modestly increased in young mice relative to the NE/10 RBD group, IFN-γ was increased by an average of 6-fold in the cLN compared to the NE/10 RBD group (Figs. 4a, 5a). Surprisingly, IFN-γ production was enhanced by an even greater degree in aged mice immunized with NE/IVT/10 RBD, resulting in average increases of 390- and 14-fold in the spleen and cLN, respectively, relative to the aged NE/10 RBD group. Similarly high IFN-γ levels were induced at the high RBD dose with NE/IVT in both young and aged groups. IM Advx/10 RBD induced similar levels of IFN-γ production as NE/IVT/10 RBD given through the IN route in splenocytes from young mice, however Advx induced notably less IFN-γ in the cLN of young mice (reduced by an average of 4-fold relative to NE/IVT/10 RBD). These results are in accordance with the more T_H_2-bias of the Addavax adjuvant. Importantly, no detectable IFN-γ was induced in aged mice immunized with Advx/10 RBD, suggesting reduced efficacy of the Advx adjuvant in the context of aging. A similar pattern was observed with the other T_H_1-associated cytokines, IL-2 and IP-10. NE/IVT/10 RBD significantly increased production of IL-2 compared to NE/10 RBD, inducing similar levels in both young and aged groups, while Advx showed significantly reduced levels of IL-2 in aged animals (Figs. 4b, 5b). NE/IVT similarly enhanced IP-10 levels relative to NE alone in aged mice, however differences were small within the spleen and more clearly observable in the cLN (Figs. 4c, 5c). Thus, for these T_H_1 cytokines, NE/IVT/10 RBD induced similar or higher levels of cytokine secretion in aged, immunized mice as in young, immunized mice, which was in contrast to the consistent reduction observed with IM Advx/10 RBD in aged mice. To further examine the contribution from antigen-specific CD8^+^ T cells, splenocytes from aged mice immunized with NE, NE/IVT, or Advx with 10 μg RBD were compared for IFN-γ production by ELISpot in response to stimulation with the H-2K^b^ class-I restricted peptide (VVLSFELL, the only identified class I mouse epitope within the RBD) (Supplementary Fig. 3)^33^. ELISpot analysis revealed a robust antigen-specific CD8^+^ T cell response with the NE/IVT which was enhanced compared to the NE alone.

Further, NE/IVT/10 RBD enhanced levels of TNF-α production in the cLN as compared to the NE and Addavax adjuvants, which induced only low levels (Fig. 5d). Interestingly, NE/IVT/10 RBD and NE/IVT/20 RBD elicited higher levels of TNF-α in aged mice as compared to young mice. Such marked enhancement of T_H_1-associated cytokines along with TNF-α production induced by the combined adjuvant in both young and aged mice is promising, as co-production of IFN-γ, IL-2, and TNF-α on polyfunctional antigen-specific T-cells has been shown to be a strong predictor of effective T-cell mediated protection against viral infection. Moreover, several studies have suggested that the reduced capacity of the aging human immune system for antiviral defense is due in part to reduced IFN-γ and IL-2 production, and a progressive shift towards a more T_H_2 bias as the immune system ages^34,35^. Thus, an adjuvant capable of inducing these strong T_H_1-biased responses in the context of aging may be able to overcome these deficiencies associated with immunosenescence.

For T_H_2-related cytokines, minimal IL-4 was induced in any of the immunized groups (Figs. 4e, 5e). While the combined adjuvant induced higher IL-4 in splenocytes from aged mice than the NE alone or Addavax, levels remained low ( < 38 pg/mL in stimulated cell supernatants) and were also low in the cLN for both age groups ( < 12 pg/mL in stimulated cell supernatants). In contrast to the pattern observed with T_H_1 cytokines, IM Advx/10 RBD elicited the most robust IL-5 production of all immunized groups in the spleen which was equal in magnitude between young and aged mice (average 487 pg/mL, 470 pg/mL, respectively), whereas NE and NE/IVT with the same RBD dose elicited minimal IL-5 in the spleen. Interestingly, Advx/10 RBD elicited even greater levels of IL-5 in the cLN of young mice (average 2,301 pg/mL), which was 4-fold higher than that elicited by NE/IVT/10 RBD (Figs. 4f, 5f). These levels were significantly lower in the cLN of aged mice given Advx/10 RBD (485 pg/mL). The high levels of IL-5 produced in the cLN for Advx are notable, as the cLN are not the draining lymph nodes for IM immunization. Relatively low levels of IL-13 were induced in the spleen or cLN across all of the young treatment groups (Figs. 4g, 5g). However, IL-13 was elevated for young mice immunized with Advx/10 RBD, consistent with its T_H_2 bias. In light of concerns over T_H_2 associated immunopathology with certain vaccines for respiratory viruses (vaccine associated enhanced respiratory disease (VAERD)), these levels of IL-13 produced concomitantly with such highly elevated IL-5 may make Addavax (MF59) a less optimal choice for an adjuvant tailored for SARS-CoV-2. Interestingly, while NE/IVT induced minimal IL-13 in the spleen or cLN of young mice, higher levels were observed in aged mice, although these levels remained relatively low and did not occur in the context of highly elevated IL-5. NE/IVT also significantly enhanced IL-6 levels relative to NE alone, inducing high levels of IL-6 particularly at the high RBD dose (Figs. 4h, 5h). Interestingly IL-6 levels were also increased in the spleen and cLN of aged mice for all adjuvanted groups as compared to the corresponding young groups. However, minimal induction of IL-6 was observed in the cLN for NE/10 RBD and IM Advx/10 RBD in either age group.

Finally, a clear synergistic effect was observed with NE/IVT in the induction of IL-17A in the spleen and cLN, with similar levels induced in both age cohorts (Figs. 4i, 5i). IN NE/IVT/10 RBD elicited an average increase in IL-17A of 19- and 21-fold relative to the IN NE/10 RBD group in the spleen and cLN of young mice, respectively, and an average increase of 75- and 18-fold in the spleen and cLN of aged mice, respectively. While no significant IL-17A was detected in the spleen or cLN for the NE alone with 10 μg RBD, increasing the antigen dose to 20 μg resulted in significant levels of IL-17A induction both in the spleen and cLN, which were further enhanced by the combined adjuvant at this antigen dose. In contrast, parenteral immunization with Advx/10 RBD did not elicit IL-17A in either the spleen or cLN. These results are consistent with our previous reports demonstrating that T_H_17 induction is specific to the mucosal route of immunization with NE. T_H_17 responses have been demonstrated to be a central component of effective host defense to viral infection, particularly at mucosal surfaces of the respiratory tract. Thus, being able to induce robust T_H_17 responses along with strong T_H_1 biased responses particularly in the context of aging with NE/IVT may provide a powerful tool for driving more potent and tailored immune responses towards SARS-CoV-2. While IL-17A has a critical role in immunoprotective mechanisms, it has also been associated with pathology in certain contexts^36^. However, it has been shown to be protective and non-pathogenic in the context of IL-10 co-production. Indeed, significant IL-10 was elicited in the spleen by NE/IVT/10 RBD in both young and aged groups which was further enhanced at the higher RBD dose (Figs. 4j, 5j). Interestingly, IM Advx/10 RBD elicited higher levels of IL-10 in the spleen than NE or NE/IVT at the same antigen dose in young mice but not in aged mice. In contrast, marked enhancement of IL-10 was observed with the NE/IVT in the cLN as compared to the NE (or Advx groups), resulting in averages of 16-fold and 8-fold increases relative to the NE alone at the 10 μg RBD dose in young and aged mice, respectively. Similarly high levels were induced by the NE/IVT/20 RBD in both age cohorts.

### Intranasal immunization with NE/IVT/RBD induces protective immune responses in aged to senescent mice reducing morbidity upon direct challenge

**Fig. 6:**
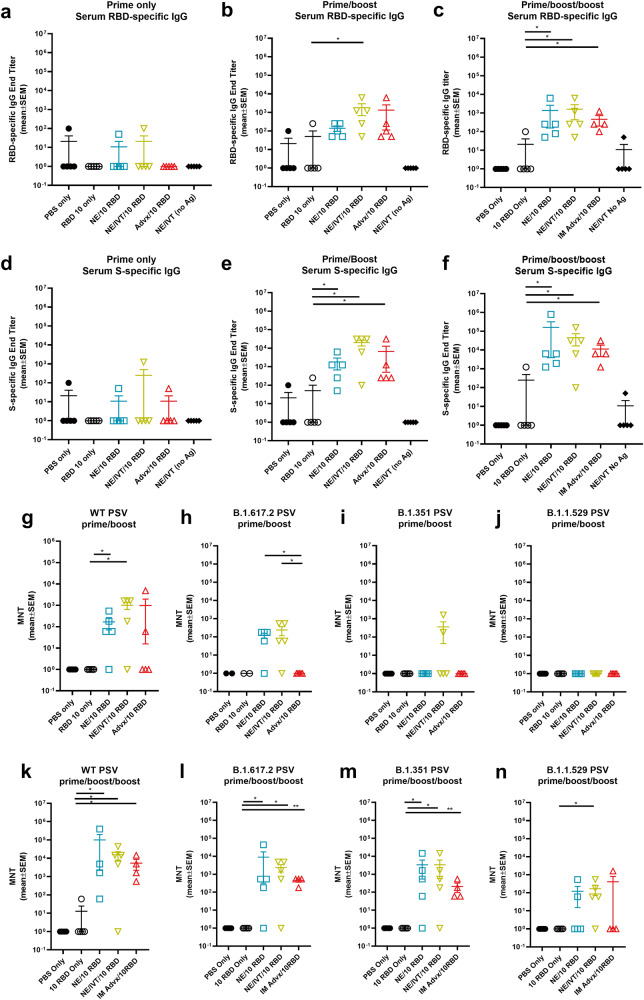
Humoral immune responses induced in aged to senescent mice. Old mice (14–17 m.o. at priming) were immunized IN with 10 μg RBD with PBS, NE, or NE/IVT, or IM with RBD with Addavax. Controls included mice immunized IN with PBS or NE/IVT without antigen. Mice were given three immunizations at a 3-week interval. Serum (**a**–**c**) RBD- and **d**–**f** S protein-specific IgG was measured 2 weeks after (**a**, **d)** prime (**b**, **e**) prime/boost and **c**, **f** prime/boost/boost immunizations. Neutralizing antibody titers were measured in sera after (**g**–**j**) prime/boost and **d**–**n** prime/boost/boost immunizations with SARS-CoV-2 PSVs for (**g**, **k**) WT, **h**, **l** B.1.617.2, **i**, **m** B.1.351, **j**, **n** B.1.1.529 variants. Titers are shown as mean ± SEM (*n* = 5/grp) (*p < 0.05, **p < 0.01 by Mann–Whitney *U* test shown between adjuvanted groups).

While the impact of aging on the induced immune response profiles could already be observed in 8 m.o. aged mice, to examine these effects in further senescent mice, 14–17 m.o. (equally distributed amongst groups) C57Bl/6 mice (at initiation, 16–19 m.o. at challenge) were immunized IN with the less optimal low 10 μg dose of RBD with PBS, NE, or NE/IVT, or IM with Addavax. Mice were again immunized according to a prime/boost/boost schedule. Serum RBD- (Fig. 6a–c) and S-specific (Fig. 6d–f) IgG was measured 2 weeks after each immunization. Total antigen-specific IgG titers induced by each of these treatment groups showed the same relative pattern as in the aged 8 m.o. mice (Fig. 1a–d and Supplementary Fig. 1A, B), with minimal induction of IgG induced after the prime in all groups, and with IN NE/IVT inducing the highest levels of RBD- and S-specific IgG after the prime/boost immunizations compared to IN NE and IM Addavax groups. The magnitude of IgG induced after the prime/boost immunizations in the 14–17 m.o. mice was also the same for each of the adjuvanted groups as what was observed with the immunized 8 m.o. aged mice. After the third immunization, this relative pattern was maintained. However, IgG titers did not appear to be boosted significantly after the third immunization for IN NE, IN NE/IVT, or IM Advx groups in the 14–17 m.o. mice as compared to the 8 m.o. mice, in which they were boosted an additional 1-2 logs, reflecting further suboptimal immune responses in these older mice. IgG subclass profiles in these senescent immunized mice were also consistent with those in the 8 m.o. aged mice (Supplementary Fig. 4).

Interestingly, while the IgG titers were not significantly boosted by the third immunization, comparison of nAb titers across WT, B.1.617.2, B.1.351, and B.1.1.529 variants after the second (Fig. 6g–j) and third immunizations, showed a significant enhancement in antibody quality after the third immunization in these aged mice (Fig. 6k–n). After two immunizations, the majority of mice in the IM Advx/10 RBD group showed no nAbs against the WT virus, and none of these mice had nAbs against the other variants. In contrast, IN NE/IVT had significant nAbs against WT virus in the majority of immunized animals at this time point as well as against B.1.617.2, with some mice having nAbs against B.1.351, and none having nAbs against B.1.1.529. After the third immunization, nAb titers were boosted for all adjuvanted groups. nAbs induced by IN NE/IVT groups were increased by approximately 1 log and showed robust neutralization across the WT, B.1.617.2, and B.1.351 variants, as well as low, but significant levels of nAbs against B.1.1.529. The relative pattern was again maintained as with the 8 m.o. mice (Fig. 2) in which IN NE/IVT induced the highest nAbs consistently across variants as compared to the Advx group, confirming the improvements in immune response with IN NE/IVT immunization.

**Fig. 7:**
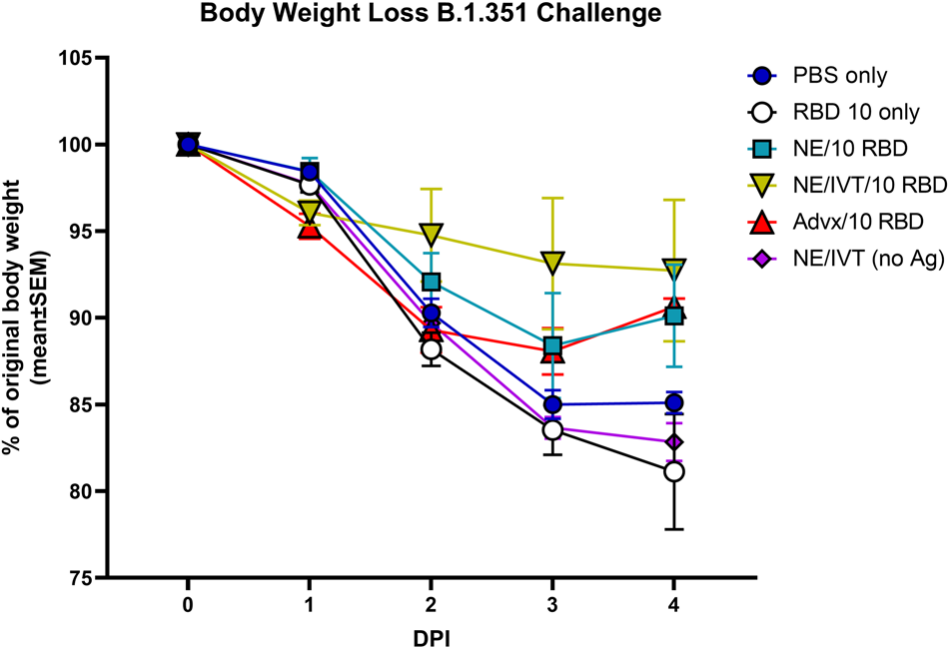
Evaluation of protection from heterologous viral challenge in senescent vaccinated mice. Aged mice (14–17 m.o. at priming, 16–19 m.o. at challenge) were immunized three times IN with 10 μg RBD with PBS, NE, or NE/IVT, or IM with RBD with Addavax. Controls included mice immunized IN with PBS or NE/IVT with no antigen. Mice were then challenged intranasally with 1 × 10^4^ pfu of B.1.351 SARS-CoV-2 three weeks after the final immunization. Average percentage body weight loss in each treatment group was recorded over the course of 4 days post-infection (d.p.i.) (shown as mean ± SEM (*n* = 5/grp)).

To evaluate the cross-protective efficacy of these adjuvants in the senescent mice, we then directly challenged the vaccinated mice intranasally with 1 × 10^4^ pfu B.1.351, three weeks after the third immunization. Morbidity was measured as a function of body weight loss over the course of 4 d.p.i. (Fig. 7). The IN RBD 10 only and IN NE/IVT alone (no antigen) groups showed the same weight-loss as the mock immunized IN PBS group, resulting in a weight loss of 15-20% by 4 d.p.i without signs of weight recovery by the end point. Of note, we have previously observed that the same viral challenge in young C57Bl/6 mice resulted in no observable weight-loss, reflecting the greater morbidity in the senescent animals. IN NE/10 RBD and IM Advx/10 RBD vaccinated animals had similar traces for weight-loss, showing less weight loss than the control groups, with a maximum weight-loss of ~13% at 3 d.p.i with weight recovery beginning by 4 d.p.i., reflecting a degree of protection. In contrast, IN NE/IVT/10 RBD offered the greatest protection, showing minimal weight-loss upon infection. The average maximum loss was ~8%, however, the majority of animals in this group (3/5) showed no weight-loss upon challenge, confirming the most optimal protection with the IN NE/IVT adjuvant.

### Immunization with NE and NE/IVT adjuvants induce durable humoral immune responses

**Fig. 8:**
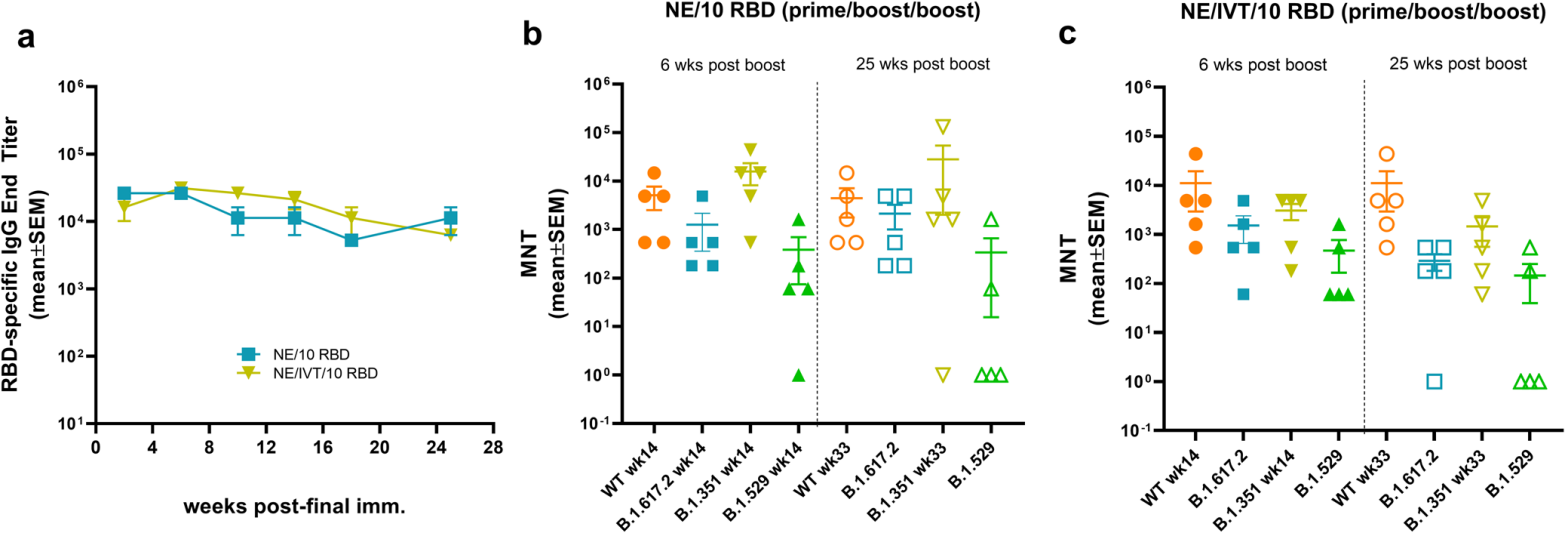
Longevity of humoral immune responses induced by IN immunization. The longevity of the induced humoral immune responses was measured in 8-week old mice given three IN immunizations at a 4-week interval with 10 μg RBD with either NE or NE/IVT. **a** Serum RBD-specific IgG titers were followed for 25 weeks after the last boost. Neutralizing antibody titers in sera were compared between the (**b**) NE/10 RBD, or (**c**) NE/IVT/10 RBD groups 6 or 25 weeks after the final immunization. Titers were measured against WT, B.1.617.2, B.1.351, and B.1.1.529 SARS-CoV-2 variants using lentivirus pseudoviruses. (data shown as mean ± SEM (*n* = 5/grp)); (*p < 0.05, **p < 0.01 by Mann–Whitney *U* test shown in Supplementary Table 1).

We next examined the longevity of the antigen-specific antibody responses induced by the NE and NE/IVT adjuvants using the 10 μg RBD antigen dose. 8-week old mice were given three IN immunizations with either NE/10 RBD, or NE/IVT/10 RBD at a 4-week interval as above, and serum RBD-specific IgG titers were measured over the course of 25 weeks after the last immunization (up to week 33 post-initial immunization) (Fig. 8a). RBD-specific IgG titers remained high and relatively constant in both the NE and NE/IVT groups, showing minimal or no reduction over the course of 25 weeks after the last immunization. Additionally, no significant changes in nAb titers were observed for either adjuvanted group against the WT, B.1.617.2, and B.1.351 variants (Fig. 8b, c). However, while 4/5 mice in the NE group and 5/5 mice in the NE/IVT group had detectable nAb titers against the B.1.1.529 variant after 6 weeks, after 25 weeks, only 2/5 mice in either group still had detectable nAb titers, suggesting loss of these low affinity nAbs. nAb titers maintained the same relative pattern of efficacy against WT, B.1.617.2, B.1.351, and B.1.1.529 at 25 weeks as at 6 weeks following the final immunization, with B.1.1.529 displaying the lowest degree of cross-neutralization. These results demonstrate that NE and NE/IVT both induce robust and long-lived protective humoral immune responses and suggest that immunization strategies with better antigen selection/design will likely lead to long-lived cross-neutralization against the B.1.1.529/BA.1 variant as well.

### IN Immunization with RBD adjuvanted with NE and NE/IVT induces long-lived antigen specific cellular immune responses

**Fig. 9:**
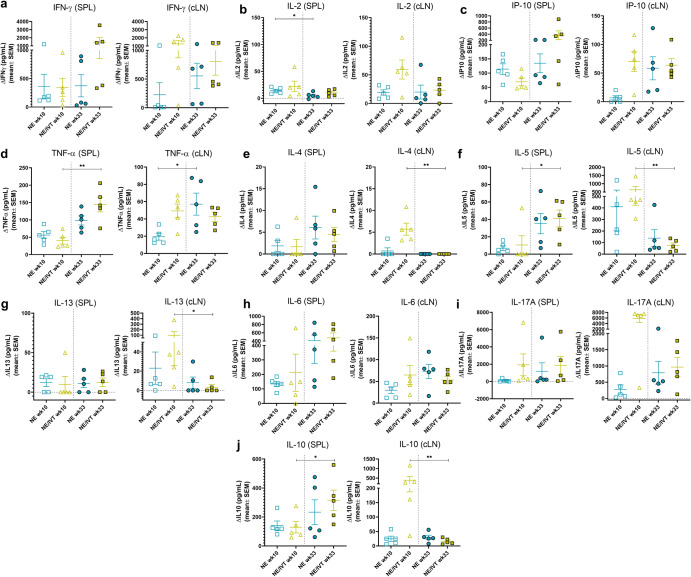
Longevity of cellular immune responses induced by IN immunization. The durability of the antigen recall responses induced by immunization was measured by comparing the levels of secreted cytokines from splenocytes (SPL) or cervical lymph node isolates (cLN) from immunized mice in response to ex vivo stimulation with 5 μg RBD for 72 h. SPL and cLN were isolated from young (8-week old) mice given three IN immunizations with 10 μg RBD with either NE or NE/IVT either 2 or 25 weeks after the last immunization (week 10 or week 33, respectively). Levels of secreted cytokines were measured in the cell supernatant by multiplex immunoassay relative to unstimulated cells for (**a**) IFN-γ, **b** IL-2, **c** IP-10, **d** TNF-α, **e** IL-4, **f** IL-5, **g** IL-13, **h** IL-6, **i** IL-17A, and **j** IL-10. (data shown as mean ± SEM (*n* = 5/grp)); (*p < 0.05, **p < 0.01 as compared between week 10 and week 33 for each adjuvant group by Mann–Whitney *U* test).

To examine the durability of the induced T cell responses, antigen recall responses were compared in splenocytes and cLN isolates from mice immunized three times IN with NE/10 RBD or NE/IVT/10 RBD at week 10 (2 weeks post-final immunization) or week 33 (25 weeks post-final immunization). Splenocytes and cLN isolates were stimulated with 5 μg RBD for 72 h, and levels of secreted cytokines were measured in the cell supernatant. Overall, the relative pattern of cytokine production was conserved over 6 months after the final immunization, with the combined NE/IVT adjuvant still inducing high levels of IFNγ, IL-2, and IP-10 at week 33 in the spleen and cLN (Fig. 9a–c). The magnitude of induction of T_H_1-associated cytokines also remained similar, if not increased at the later time point. For example, IFN-γ levels appeared to increase substantially in the spleen in some mice immunized with NE/IVT 6 months after the final immunization relative to those isolated two weeks after the final immunization (giving an average overall increase of 4-fold, and by as high as 47-fold (Fig. 9a). Similar increases in IP-10 and TNF-α were observed in the spleen for both NE and NE/IVT immunized mice at 6 months versus two weeks, as well as in the cLN for the NE alone group (Fig. 9c, d). Such maintained (or enhanced) production of T_H_1-associated cytokines and TNF-α over 6 months supports the durability of NE/IVT-induced anti-viral T-cell responses.

As with the early T-cell responses, minimal IL-4 was observed upon RBD stimulation at week 33 in the spleen and no detectable IL-4 was observed in the cLN of NE or NE/IVT immunized mice (Fig. 9e). Interestingly, while IL-5 appeared modestly increased at week 33 in the spleen for NE and NE/IVT groups, levels remained low <70 pg/mL, and were markedly reduced in both groups in the cLN at week 33 relative to week 10 (Fig. 9f). Similarly, IL-13 remained low in the spleen and were reduced to undetectable levels in the cLN at week 33 (Fig. 9g). Thus, while T_H_1-associated cytokines were maintained or enhanced over 6 months, T_H_2-associated cytokines were maintained in the spleen but were consistently reduced in the cLN. In contrast, IL-6 levels showed a similar pattern as the T_H_1 cytokines, with IL-6 in the spleen increasing after 6 months in both NE and NE/IVT groups, while cLN levels were unchanged (Fig. 9h).

Finally, comparison of IL-17A levels at week 10 and week 33 showed maintenance of the high levels of IL-17A produced in the spleen in response to antigen stimulation, with increased production of IL-17A by the combined adjuvant relative to the NE alone (Fig. 9i). In contrast, while IL-17A in the cLN of NE/IVT immunized mice at week 33 were still high and enhanced compared to the NE alone, overall levels in the cLN were notably lower at the late time point (mean 5839 pg/mL, range 332–7525 pg/mL at week 10; mean 965 pg/mL range 126–1777 pg/mL at week 33 for NE/IVT). Significant increases in IL-10 were also observed at the late time point in the spleen, especially for the NE/IVT immunized group, whereas a marked drop in IL-10 production in the cLN was observed (Fig. 9j). The analogous pattern for IL-17A and IL-10 could potentially suggest these cytokines are produced by the same non-pathogenic T_H_17 cells. Taken together, these results demonstrate that IN immunization with NE/IVT induces long-lived T cell responses, which are potentially more enhanced over time towards a greater T_H_1/T_H_17 bias with less induction of T_H_2 cytokines.

## Discussion

We have previously shown the benefit of targeting multiple innate immune pathways with the intranasal combined adjuvant, NE/IVT, on the outcome of vaccination approaches for influenza and COVID-19 in young mice^15,16^. Here, we build further on our previous insights to investigate the benefit of the NE/IVT on vaccine efficiency in aged mice and address the longevity of vaccine-induced immune responses. Recombinant monomeric SARS-CoV-2 RBD was used as test antigen, as it is the main target for nAbs, and because it is less immunogenic than the full-length spike protein, allowing for better comparisons of efficacy between adjuvants using a minimally protective antigen. RBD was tested in two doses: 10 μg in both young and aged (8 m.o.) mice for all adjuvant groups, and 20 μg in select groups (with NE and NE/IVT in young mice or with NE/IVT in aged mice) to examine dose effect. NE and NE/IVT induced robust RBD-specific IgG in young and aged mice, with the NE/IVT resulting in the highest titers, followed by NE when compared at the same antigen dose. Both IN NE and IN NE/IVT outperformed IM Addavax for the induction of total RBD-specific IgG in both young and aged animals at the same antigen dose. Serum nAbs have been well established as strong correlates of protection against COVID-19. Both NE and NE/IVT induced high levels of nAbs against WT virus that were higher than Addavax in both young and older animals when compared at the 10 μg RBD dose. Importantly, while NE and NE/IVT immunization groups maintained similar levels of induction of nAbs against WT virus in aged mice as in young, nAb titers induced by Addavax in aged 8 m.o. mice were reduced compared to young. These differences between the IN adjuvants and Addavax in young and aged mice were particularly evident upon evaluation of cross-neutralizing antibodies against divergent variants. NE and NE/IVT both induced higher nAbs against B.1.617.2 and B.1.351 and showed better maintenance of these titers in aged mice compared to Addavax. These results support induction of better-quality antibody responses by the IN adjuvants and suggest that immune responses potentiated by Addavax are more affected by age of the vaccinated host compared to NE or NE/IVT. NE and NE/IVT were also more effective at inducing nAbs against the B.1.1.529 Omicron variant in young mice with 10 μg RBD than Addavax. These nAb titers were, however, reduced in aged 8 m.o. mice. Since VOCs have acquired mutations in the RBD that allow the virus to escape from nAbs, it is important to maximize antibody responses by vaccination. As such, a drop in neutralization will not leave the vaccinated host unprotected when nAb titers are still high enough, albeit with reduced efficiency. In this regard, the B.1.1.529 variant has acquired the most mutations in the RBD amongst all of the VOCs evaluated, and escape from nAbs has been reported for human vaccinees, similar to what was observed in this experiment^37^. However, nAbs across all VOCs evaluated, including B.1.1.529 were equally high in both young and aged animals immunized IN with 20 μg RBD + NE/IVT, further demonstrating the ability to induce equally potent cross-neutralizing antibodies to more divergent VOCs in young and aged animals with NE/IVT at a higher antigen dose. Thus, better protection against VOCs across age cohorts may be obtained through employing more potent adjuvants such as NE/IVT. These results are highly significant, as MF59 (Addavax equivalent) is the adjuvant currently licensed for use in influenza vaccines for the elderly (Fluad®), and thus highlight the potential for improving vaccine responses in the context of aging with the IN NE/IVT adjuvant.

To correlate the humoral vaccine responses with protection, sera from mice vaccinated with 20 μg RBD were transferred into naive young receiver mice, followed by challenge with MA-SARS-CoV-2 or B.1.351. Both viruses contain the RBD N501Y mutation, and therefore can directly infect WT mice. Whereas we and others have shown previously that this mutation does not seem to affect neutralization by sera induced by vaccination with ancestral (Wuhan-like) vaccines, other key mutations present in the RBD of B.1.351 (K417N and E484K) have a significant impact on neutralization by post-vaccination sera^31^. We opted to use SARS-CoV-2 challenge models with WT mice for these studies rather than the K18-hACE2 transgenic mouse model, since morbidity upon experimental SARS-CoV-2 infection in the latter mouse model is to a large extent from direct brain infection due to high exogenous expression of hACE2 in the brain. As such, this does not accurately represent human disease progression during COVID-19^38,39^. Moreover, the MA-SARS-CoV-2 and B.1.351 challenge models in WT mice offer the advantage of allowing study of infection localized primarily to the respiratory tract. Sera from mice vaccinated with NE/IVT/RBD gave the largest reduction of lung viral titers by 3 d.p.i. for both MA-SARS-CoV-2 and B.1.351 viruses, as compared to sera from mice vaccinated with NE/RBD or RBD alone. Furthermore, sera from both young and aged donor mice vaccinated with NE/IVT/RBD resulted in a similar magnitude of lung viral titer reduction towards both viruses in receiver mice. It is possible that the observed variation in lung viral titers for NE/IVT/RBD young and aged groups suggests that in mice with full lung virus control, a critical threshold level of nAbs was reached, whereas this was not the case in mice with detectable virus titers. Of note, these sera had equivalent WT nAb titers. These results suggest that antibody-mediated protection involves activities beyond simple virus neutralization, such as ADCC, ADCP and complement activation which may also contribute to virus control in vivo. While nAbs and non-neutralizing antibodies are critical for protection, it has been established that a combination of nAbs and robust T cell responses are key to complete protection from infection. The optimal cytokine profile induced by NE/IVT as discussed below, tailored towards a T_H_1/T_H_17 response combined with the efficacy of the induced nAbs support robust protection in both young and aged mice.

Several studies have suggested that T cell functionality is reduced in aged individuals through either reduction in naïve T cell reservoirs, less responsiveness of these naïve T cells in the elderly, and/or preferential T_H_2 polarization^34^. Defects in IFN-γ and IL-2 production with age have been shown to correlate with reduced protection from viral infection and reduced vaccine efficacy. Recent studies demonstrate that decreased IFN-γ and IL-2 production correlates with reduced protection from SARS-CoV-2 infection after mRNA vaccination, especially in the elderly^35^. As a RIG-I agonist, IVT DI is a potent inducer of IFN-Is, thereby promoting strong T_H_1-biased cellular responses^24,25^. Accordingly, we observed that NE/IVT results in the strongest induction of type 1-skewed T cell responses as reflected in the cytokine profiling of antigen-specific T cell recall responses measured in splenocytes and cells derived from the cervical lymph nodes (draining the nasal mucosa) from immunized animals. Marked enhancements of IFN-γ, IL-2, and IP-10 production were observed in the antigen-stimulated splenocytes and cLN isolates from both young and aged NE/IVT immunized mice. Surprisingly, equivalent, or even higher levels of induction of these cytokines were observed with NE/IVT in aged mice compared to young. These findings suggest that the combined adjuvant may be able to overcome some of these deficiencies associated with immunosenescence. Further, polyfunctional T cells producing IFN-γ, IL-2, and TNF-α are a strong marker of optimal effector function in response to vaccination, and these three cytokines are strongly enhanced by NE/IVT in both age cohorts, suggesting induction of better quality T cell responses^40^. Finally, a comparison of IFN-γ production in splenocytes from aged mice immunized with NE and NE/IVT, stimulated with an H-2K^b^ restricted peptide by ELISpot analysis revealed a significant contribution from antigen-specific CD8^+^ T cell response with the combined adjuvant (Supplementary Fig. 3). Promotion of T_H_1 responses by NE/IVT was also reflected in the IgG subclass profile post-vaccination and was equivalent in both young and aged mice. In line with our previous findings, compared to NE alone, NE/IVT was also a strong inducer of IL-17A in combination with IL-10, which has been shown to contribute to protection during mucosal infections. Our previous work has demonstrated that this T_H_17 response is unique to the IN route of immunization with the NE adjuvant. Addavax, on the contrary, showed a more type 2-skewed vaccine response compared to either NE or NE/IVT, characterized by a more IgG1-driven antibody response and absence of strong T_H_1 or T_H_17 cytokines, but with typical T_H_2 associated cytokines IL-4 in aged mice and IL-5 in both young and aged mice upon restimulation.

Since our NE/IVT adjuvant is developed for mucosal administration, we also measured mucosal antigen-specific IgA in BAL fluid after the second booster vaccination and found that NE/IVT outperformed NE and Addavax in both young and aged mice, which did not induce detectable IgA at the low RBD dose. Moreover, an antigen dose effect on mucosal IgA levels was observed for mice that received 20 μg of RBD + NE/IVT, which was not as apparent for NE alone in young animals, illustrating that the addition of IVT can help enhance the mucosal immune response to recombinant protein antigens. Notably, NE alone typically induces high mucosal IgA production with other antigens tested^16,41^. However, the low levels of IgA induced by NE alone in this study likely reflects the low immunogenicity of the RBD antigen. Induction of potent antibody responses at mucosal surfaces, the port of entry for respiratory viruses, by vaccination is required to provide optimal protection and prevent transmission. However, inducing mucosal immune responses with recombinant protein antigens typically requires strong innate immune activation by adjuvants to overcome tolerance at mucosal sites. Thus, the ability of NE/IVT to safely induce mucosal IgA with a poorly immunogenic antigen and further highlights the benefits of this mucosal adjuvant. In addition to IgA, high levels of RBD-specific IgG were detected in the BAL for the combined adjuvant groups which also contribute to protection at the mucosal surface. T_H_17 cells have been shown to be critical in promoting high and sustained levels of IgA production at mucosal sites-particularly the lung, and in the establishment of resident memory T cells^42^. We have previously shown that IL-17A is a critical component of NE-mediated immunity, as IN immunization of IL-17A knock-out mice with NE resulted in severely attenuated nAb responses and no IgA induction in influenza vaccination models (unpublished data). The significant enhancement of IL-17A production observed in NE/IVT immunized mice support robust induction of T_H_17 cells and are consistent with the observed IgA profile.

While changes in immune response induction and quality between these adjuvants with age could already be observed in 8 m.o. aged mice, to further investigate the impact of age on these observed differences, we confirmed our findings in even older mice (14–17 m.o. at initiation, 16–19 m.o. at completion) as a model representing a greater degree of immunosenescence. Mice immunized with the lower 10 μg suboptimal dose of RBD IN with NE and NE/IVT or IM with Addavax showed similar magnitudes of induced RBD- and S- specific IgG in the 14–17 m.o. mice as observed in the 8 m.o. mice after prime/boost immunizations but showed less of a boost effect after the third immunization as seen in the 8 m.o. animals. This reflects the less overall robustness of the immune responses in these aged animals. Nonetheless, nAb titers induced in the 14–17 m.o. mice remained equivalent to those induced in the 8 m.o. aged animals, with IN NE/IVT induced the highest cross-variant nAbs compared to IN NE and IM Advx. These differences between adjuvants were correlated with protection from direct challenge with the heterologous B.1.351 variant, in which IN NE/IVT provided greater protection from morbidity as compared to the IN NE or IM Advx groups.

Lastly, we characterized the longevity of the vaccine-induced immune responses in mice that were immunized when young with 10 μg RBD, either unadjuvanted or adjuvanted with NE or NE/IVT. From our experience with influenza vaccine validation in mouse models, we are aware of the fact that longevity of humoral immune responses in the mouse model does not always reflect the human situation. Antibody titers induced in young immunologically naive animals by vaccination often remain high for a prolonged period, whereas this is not always the case in humans. Indeed, it is observed that full-length spike protein- and RBD-specific antibody titers wane over time in human vaccinees and booster immunizations have been required to maintain protective nAb titers. Serum IgG titers against RBD and nAbs against VOCs induced by NE/IVT immunization remained constant for at least 25 weeks post-final vaccination. Only nAbs against B.1.1.529 appeared to be affected to some extent over time, which may reflect the lower affinity of the induced antibodies. However, such optimal maintenance of nAb titers to the other VOCs tested suggests that durable nAb titers can be achieved with NE/IVT towards B.1.1.529 with strategic antigen design beyond the RBD alone. Upon evaluating the longevity of T cell responses in both spleen and cLN, we found that the protective T_H_1/T_H_17 profile induced by NE/IVT was also maintained over the 6-month period, and this despite RBD being a relatively small antigen, and therefore harboring only a limited number of potential T cell epitopes. The importance of inducing memory T cell responses by vaccination for protection against SARS-CoV-2 infection across variants has become clear as nAbs lose potency as novel variants emerge. Memory effector T cells may not only remove infected cells, they may also speed up de novo or recall B cell responses as first responders during infection by providing immediate bystander help. Such robust and durable T_H_1/T_H_17 responses induced by NE/IVT make it a highly promising adjuvant for long-term protection as new variants emerge.

Other groups have also demonstrated promise with IN immunization against SARS-CoV-2. Much interest has focused on adenovirus vectored IN vaccines, for example, a chimp-adenovirus vectored S1/nucleocapsid/RdRp construct and an adenovirus-type 5 vectored RBD^43,44^. Both constructs were highly effective at inducing mucosal IgA after a single dose with induction of airway memory T cells. However, both showed minimal or no induction of antigen-specific T cell responses in the spleen, which were short-lived (<14 days). RBD has shown potential as a vaccine antigen in different animal models and has been in clinical trials as a vaccine candidate, albeit as a dimer rather than a monomer, most likely because of the poor intrinsic immunogenicity of the RBD^45,46^. An IN adjuvanted recombinant RBD vaccine has been tested in preclinical animal models, however, the adjuvant was polyethyleneimine, a cationic lipid. Polycationic lipids are an interesting avenue that is being explored for mucosal adjuvants, but need to be modified and carefully tested to reduce reactogenicity at mucosal sites particularly where there is possibility for unintended delivery to the lung, due to interactions with phospholipid cellular membranes. This is expected to be an important field of future research given their inclusion in lipid nanoparticles for mRNA vaccines as well.

While mouse models of aging cannot fully recapitulate human immunosenescence, much of our understanding of aging of the human immune system and vaccine responses in the elderly has been gained from preclinical mouse models^47–50^. Taken together, we have shown that the combination of NE and IVT results in a superior and balanced long-lived T_H_1/T_H_17-driven vaccine response when compared to the single NE adjuvant or Addavax. This vaccine response is characterized by potent induction of memory B and T cells, with type 1 class-switched antibodies that are cross-neutralizing towards multiple VOCs and correlate with protection during live virus challenge, even with SARS-CoV-2 viruses that are antigenically mismatched with the vaccine antigen. These findings combined with the ability to induce mucosal immune responses highlight the potential of NE/IVT as a promising adjuvant for more effective intranasal vaccines that can be effective in both young and aged populations.

## Methods

### Adjuvants and antigen

NE was produced by emulsifying cetylpyridinium chloride (CPC) and Tween 80 at a 1:6 (w/w) ratio, with ethanol (200 proof), super refined soybean oil (Croda) and molecular grade water using a high-speed homogenizer until a uniform particle size (*d* = 450–550 nm) and charge (zeta potential = 50–55 mV) was achieved. The sequence and synthesis of IVT DI RNA have been described in detail^15,24^. Briefly, SeV DI RNA was amplified using a 5′ primer with the T7 promoter and a 3′ primer with the hepatitis delta virus genomic ribozyme site followed by the T7 terminator and cloned into a pUC19 plasmid. IVT DI was in vitro transcribed using a HiScribe T7 High Yield RNA synthesis kit (New England Biolabs) followed by DNAse I clean-up with a TURBO DNA-free kit (Thermo-Fisher) according to the manufacturer’s protocol. IVT DI was then purified with an RNeasy purification kit (Qiagen), and the absence of endotoxin was verified by a limulus amoebocyte lysate assay (ThermoFisher). Recombinant SARS-CoV-2 receptor binding domain (RBD) (aa319-545) derived from the WT (Wuhan-Hu-1) SARS-CoV-2 isolate with a C-terminal His tag was produced in ExpiCHO cells and purified by the University of Michigan Center for Structural Biology by purification through an affinity column followed by a size exclusion column (Superdex 200)^51^.

### Cell lines

Vero E6 cells (ATCC) were maintained in DMEM supplemented with 10% heat inactivated fetal bovine serum (HI FBS) and 1X non-essential amino acids (NEAA). HEK293T cells expressing hACE2 (293T-hACE2) were obtained from BEI resources and maintained in HEK293T medium: DMEM containing 4 mM l-glutamine, 4500 mg/L glucose, 1 mM sodium pyruvate and 1500 mg/L sodium bicarbonate, supplemented with 10% HI FBS.

### Viruses

SARS-CoV-2 clinical isolate USA-WA1/2020 (BEI resources; NR-52281) (referred to as the WT virus), and the B.1.351 variant (a kind gift from Dr. Andy Pekosz) viruses were propagated by culture in Vero E6 cells. MA SARS-CoV-2: Mouse-adapted SARS-CoV-2 was obtained by serial passage of the USA-WA1/2020 clinical isolate in mice of different backgrounds over eleven passages, as well as on mACE2 expressing Vero E6 cells^31^. Briefly, the virus was passaged every two days via IN inoculation with lung homogenate-derived supernatants from infected mice. All viral stocks were verified by deep sequencing. All work with authentic SARS-CoV-2 viruses were performed in certified BSL3 or ABSL3 facilities in accordance with institutional safety and biosecurity procedures.

### Lentivirus pseudotyped virus

Pseudotyped lentiviruses (PSVs) expressing the SARS-CoV-2 spike proteins from WT, B.1.351, B.1.617.2, and B.1.1.529 (BA.1) variants were generated using a lentivirus packaging vector, psPAX2, and a plasmid carrying the envelope protein–full-length SARS-CoV-2 spike protein (aa’s 738–1254 of the WT spike) containing a C-terminal 19 amino acid deletion to remove the ER retention signal (Invivogen). These were co-transfected with a pGF1-CMV proviral plasmid into 293T cells using standard PEI transfection methods (Polysciences, Inc)^15,52^. Removal of the ER retention signal has been shown to dramatically increase the yield of spike pseudotyped lentivirus. The pGF1-CMV plasmid carries GFP and luciferase reporter genes. Supernatants were collected and pooled after 72 h, pelleted by centrifugation at 26,000 × *g* for 4 h at 4 °C, and resuspended in DMEM. Viral titers (TU/mL) across variants were determined by measuring PSV transduction of GFP in 293T-hACE2 cells. Harvested lentivirus was stored at −80 °C. Neutralization assays with these lentivirus PSVs have been demonstrated by us and numerous groups to be representative of authentic virus neutralization assays^15,53^.

### Animals

All animal procedures were approved by the Institutional Animal Care and Use Committees (IACUC) at the University of Michigan and Icahn School of Medicine at Mt. Sinai and were carried out in accordance with these guidelines. For young mice, 6–8-week-old female C57Bl/6 mice (Jackson Laboratory) were housed in specific pathogen-free conditions. Mice were acclimated for 2 weeks prior to initiation of each study. For aged mice, female C57Bl/6 mice that were 8-month old at the initiation of the study were used. For challenge studies, mice were transferred to ABSL3 facilities 2 d prior to serum transfer and/or viral challenge.

### Immunization

For intranasal (IN) immunization, mice were anesthetized under isoflurane using an IMPAC6 precision vaporizer and given 12 μL total (6 μL/nare) of each vaccination mixture. Each group received a total of three immunizations of the same formulations at 4-week intervals. 10 or 20 μg of recombinant RBD was administered alone or with either 20% NE (w/v), or 20% NE with 0.5 μg of IVT DI in PBS. For intramuscular (IM) immunization, mice were given 50% (v/v) Addavax with 10 μg of RBD in PBS in a total volume of 50 μL. Sera were obtained by saphenous vein bleeding 2 and 4 weeks after each immunization, and by cardiac puncture at the end of the experiment at week 10. Bronchial alveolar lavage (BAL) was obtained by lung lavage with 0.8 mL PBS containing protease inhibitors. Spleens and cervical lymph nodes were harvested, processed to single-cell suspensions, and cultured for antigen recall response assessment. For longevity studies, sera were obtained every 2 weeks after the last immunization for 33 weeks, after which the mice were sacrificed for T cell response analysis.

### ELISA

Immunograde 96-well ELISA plates (Midsci) were coated with 100 ng RBD in 50 μL PBS/well overnight at 4 °C, and then blocked in 200 μL of 5% non-fat dry milk/PBS for 1 h at 37 °C. Sera or BAL from immunized mice were serially diluted in PBS/0.1% BSA. Blocking buffer was removed, and diluted sera were added to the wells and incubated for 2 h at 37 °C followed by overnight incubation at 4 °C. Plates were washed with PBST (0.05% Tween20), and respective alkaline phosphatase conjugated secondary antibodies diluted in PBS/0.1% BSA were added (1:5,000 goat-anti-mouse IgG H + L (cat. 115-055-003), 1:10,000 goat-anti-mouse IgG1 (cat. 115-055-205), 1:5,000 goat-anti-mouse IgG2b (cat.115-055-207), or 1:5,000 goat-anti-mouse IgG2c (at.115-055-208) from Jackson ImmunoResearch Laboratories, or 1:2,000 rabbit-anti-mouse IgA (cat. 610-4506) from Rockland Immunochemicals). Plates were incubated at 37 °C for 1 h, washed with PBST, and then developed at RT by the addition of p-nitrophenyl phosphate (pNPP) substrate (Sigma-Aldrich). Absorbance was measured at 405 nm, and titers were determined using a cutoff value defined by the sum of the average absorbance at the lowest dilution of naïve serum and two times the standard deviation.

### Pseudovirus microneutralization (MNT) assays

9 × 10^3^ 293T-hACE2 cells were seeded overnight on white clear bottom 96-well tissue culture plates in HEK293T medium. To titer PSVs, stocks were serially diluted in HEK293T medium with 16 μg/mL polybrene (Sigma-Aldrich), incubated for 1 h at 37 °C, and then added to the 293T-hACE2 cells and incubated at 37 °C for 4 h. Infection medium was then replaced with fresh HEK293T medium without polybrene and incubated for an additional 72 h at 37 °C. Infection medium was removed, and luciferase activity was measured by addition of 25 μL PBS and 25 μL BrightGlo luminescence reagent with an injection luminometer. Cells were incubated with the BrightGlo reagent for 5 m, after which the luminescence was measured over an integration time of 1 s. A PSV titer for use in neutralization assays across variant PSVs was selected based on the titer of WT PSV which gave >100,000 RLUs above background. For microneutralization assays, 293T-hACE2 cells were seeded overnight. Sera from immunized mice were serially diluted by a factor of three, starting at a dilution of 1:50 in HEK293T medium with 16 μg/mL polybrene (Sigma-Aldrich). 50 μL of diluted sera was added to 50 μL of diluted PSVs (8325 TU/mL), incubated for 1 h at 37 °C, and then added to 293T-hACE2 cells for incubation at 37 °C for 4 h. Infection medium was removed and replaced with fresh medium without polybrene and incubated for an additional 72 h at 37 °C. Luminescence was measured with BrightGlo reagent (Promega) for all viral variants besides B.1.6.17.2, for which SteadyGlo reagent (Promega) was used. Neutralization titers were determined as the dilution at which the luminescence remained below the luminescence of the (virus only control-uninfected control)/2.

### Microneutralization Assays

MNT assays with WT SARS-CoV-2 (2019-nCoV/USA-WA1/2020) were performed in a BSL3 facility. 4 × 10^4^ Vero E6 cells were seeded per well in a 96-well tissue culture plate overnight. Serum samples were heat-inactivated for 30 m at 56 °C and serially diluted by a factor of three, starting at dilutions of 1:30 in infection medium (DMEM, 2% FBS, 1x non-essential amino acids). Diluted sera were incubated with 250xTCID50 of the WT virus ( ~ 40 PFU) for 1 h at 37 °C, and then added to the cells for 48 h at 37 °C. Cells were fixed in 4% formaldehyde, washed with PBST (0.1% Tween 20), and permeabilized with 0.1% TritonX100 for 20 m at RT. The plates were washed three times in PBST and blocked in blocking buffer (PBST + 5% non-fat milk) for 1 h at RT. Plaques were immunostained with a mixture of mouse anti-SARS-CoV-1/2 nucleoprotein (clone 1C7C7) and mouse anti-SARS-CoV-2 spike (clone 2B3E5) monoclonal antibodies (produced by the Center for Therapeutic Antibody Development at the Icahn School of Medicine at Mount Sinai) both at dilutions of 1:1,000 (2 μg/mL) each in blocking buffer for 1.5 h at RT with gentle shaking. Plaques were then incubated with an HRP-conjugated goat-anti-mouse IgG secondary antibody (Abcam clone 2B3E5 cat ab6823) at a dilution of 1:5,000 in blocking buffer for 1 h at RT. Finally, plaques were washed and developed by the addition of 100 μL KPL TrueBlue peroxidase substrate (Seracare) prior to measuring the absorbance at 450 nm. Percentage inhibition was calculated against virus only infected controls. The 50% inhibitory dilution (ID50) values were calculated for each sample by least squares fit. Samples with ID50 values lower than the limit of detection (inverse of the lowest dilution = 30) were designated as having a titer of 10^0^.

### Passive transfer and challenge

Equal volumes of serum samples collected after the first boost immunization (week 6) were pooled from mice in each immunization group, and 50 μL of the pooled sera was passively transferred into each naïve C57Bl/6 mouse through the intraperitoneal (IP) route 2 h prior to IN challenge under mild ketamine/xylazine sedation with 10^4^ PFU of MA-SARS-CoV-2 in 30 μL. Body weight changes were recorded every 24 h, and mice were sacrificed at 3 d.p.i. Lungs were harvested in 500 μL of PBS, and the homogenate was prepared for virus titration by plaque assay.

### Antigen recall response

T cell antigen recall response was assessed in cellular isolates obtained by processing the spleen and cLN of immunized mice 2 weeks after the final immunization (week 10, or week 33 for longevity studies). For antigen recall, isolated cells were plated at a density of 8 × 10^5^cells/well and stimulated with 5 μg/well RBD (WT) in T cell media (DMEM, 5% FBS, 2 mM L-glutamine, 1% NEAA, 1 mM sodium pyruvate, 10 mM MOPS, 50 μM 2-mercaptoethanol, 100 IU penicillin, and 100 μg/mL streptomycin), in a total volume of 200 μL for 72 h at 37 °C. Secreted cytokines (IFN-γ, IL-2, IP10, IL-4, IL-5, IL-6, IL-13, IL-10, IL-17A, and TNF-α) were measured relative to unstimulated cells in supernatants using a Milliplex MAP Magnetic Mouse Cytokine/Chemokine multiplex immunoassay (EMD Millipore).

### ELISpot

ELISpot assays were performed using an alkaline phosphatase mouse IFN-γ ELISpot Plus kit (Mabtech). Briefly, splenocytes isolated from mice 2 weeks after the final immunization (wk10) were plated at a density of 6.5 × 10^5^ cells/well on a coated MSIP PVDF filter plate (Millipore),and stimulated with 4 μg/mL of the H-2K^b^ class I restricted peptide, VVLSFELL (Genscript) in T cell media for 48 h. Stimulated cells were washed and spots were developed according to the manufacturer’s protocol. IFN-γ positive cells were quantified on an AID iSpot Elispot reader.

### Direct challenge

For direct challenge in senescent mice, 14–17 months old C57Bl/6 mice were randomized equally for age and given three immunizations of the same formulations according to a prime/boost/boost schedule with a three-week interval. Mice were immunized as described above IN with 10 μg of RBD with PBS, 20% NE, or 20% NE/0.5 μg of IVT DI, or IM with 10 μg of RBD with 50% (v/v). Sera were obtained by saphenous vein bleeding two weeks after each immunization. Mice were then challenged three weeks after the third immunization through the intranasal route with 1 × 10^4^ pfu of B.1.351. Body weight loss was monitored over the course of four days post-infection. Mice were 16–19 months old at the time of challenge.

### Statistical analysis

Statistical analyses for acute cytokine production, antibody titers, viral neutralization titers, post-challenge lung pfus, and T cell recall responses were performed with GraphPad Prism 9 (GraphPad Software). Comparisons between treatment groups were performed by Mann–Whitney *U* test.

### Reporting summary

Further information on research design is available in the Nature Research Reporting Summary linked to this article.

## Supplementary information

Supplementary Information

REPORTING SUMMARY

## Data Availability

All data generated and analyzed that support the findings of this study are included in this published article (and its supplementary information files).
