# Supplementary material for: Multicomponent intranasal adjuvant for mucosal and durable systemic SARS-CoV-2 immunity in young and aged mice
Source: NPJ Vaccines. 2023 Jun 29;8:96. doi: 10.1038/s41541-023-00691-1 (PMC10310740; doi:10.1038/s41541-023-00691-1)
Supplement: Supplementary file 1 — Supplementary Information [file 41541_2023_691_MOESM1_ESM.pdf]

SUPPLEMENTAL INFORMATION:

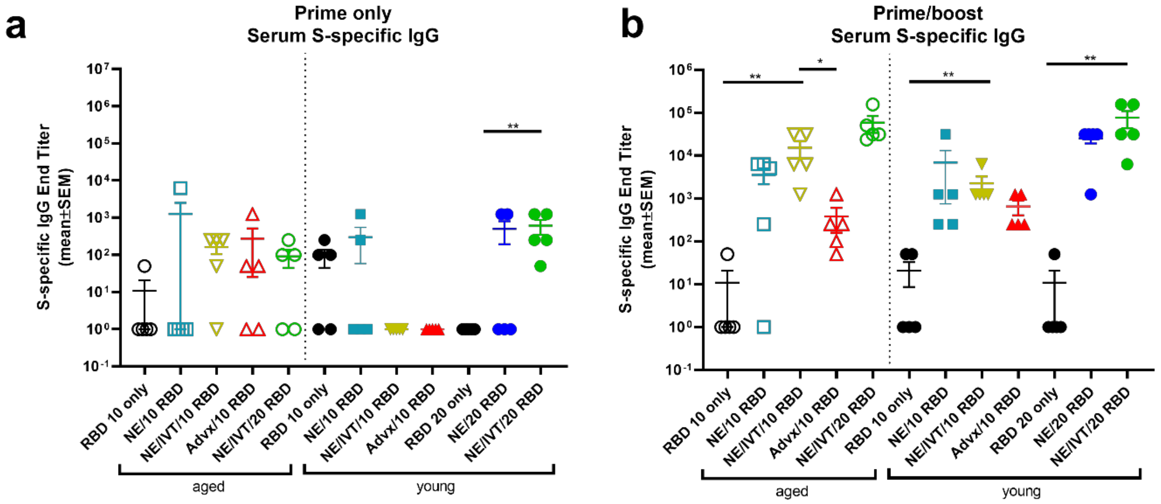

Supplementary Figure 1. Comparison of full-length S protein-specific IgG titers in immunized mice from Figure 1B-D. Young (8wk) and aged (8mo) mice were immunized IN with 10 or 20 µg RBD with PBS, NE, or NE/IVT, or IM with RBD with Addavax. S-specific IgG titers were measured in sera by ELISA two weeks after (A) prime and (B) prime/boost immunizations. Titers are shown as mean±SEM (n=5/grp) (*$p<0.05$, **$p<0.01$ by Mann-Whitney U test shown only for NE/IVT/10 RBD compared to other treatments in the same age group at the same RBD dose, and for NE/IVT/20 RBD compared only to high dose RBD groups within the young group.)

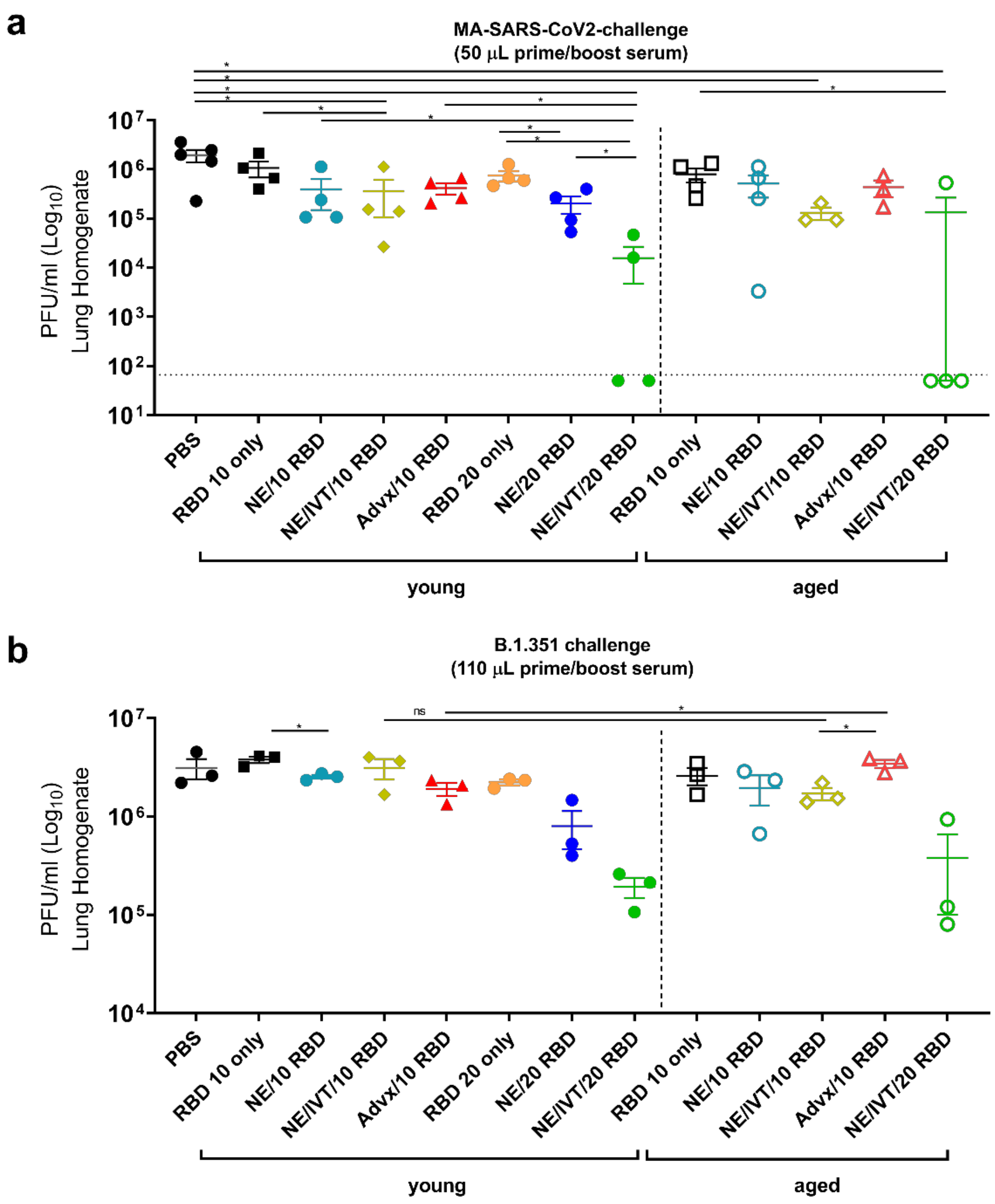

Supplementary Figure 2. Passive transfer of prime/boost serum from young and aged immunized mice into naïve mice and challenge with MA-SARS-CoV-2. (A) Sera from young or aged mice given two IN immunizations with 10 or 20 μg RBD with PBS, NE, or NE/IVT, or IM immunizations with 10 μg RBD and Addavax were pooled at wk 6, and 50 μL of the pooled serum was transferred IP into each naïve mouse 2h prior to challenge IN with 10^4 pfu MA-SARS-CoV-2. Viral load was assessed in lung homogenate by plaque assay at 3dpi. The plaque detection limit is indicated by the horizontal dashed line. Lung viral titers are shown as mean±SEM (n=5/grp) (*$p<0.05$, **$p<0.01$ by Mann-Whitney U test). **(B)** Sera from young or aged mice given two IN immunizations with 10 or 20 μg RBD with PBS, NE, or NE/IVT, or IM immunizations with 10 μg RBD and Addavax were pooled wk 10, and 110 μL of the pooled serum was transferred IP into each naïve mouse 2h prior to challenge IN with $5x10^3$ pfu B.1.351. Viral load was assessed in lung homogenate by plaque assay at 3dpi. The plaque detection limit is indicated by the horizontal dashed line. Lung viral titers are shown as mean±SEM (n=5/grp) (*$p<0.05$, **$p<0.01$ by unpaired t-test shown only for select groups of interest).

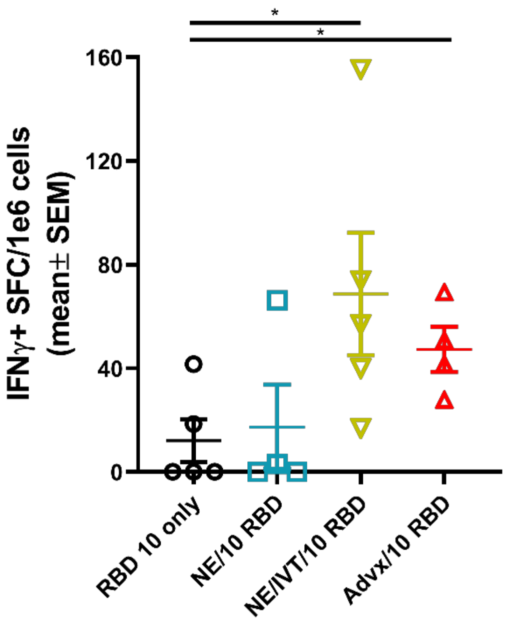

Supplementary Figure 3. ELISpot analysis of IFNγ production in antigen-specific CD8$^+$ T cells in splenocytes from aged immunized mice. Splenocytes were isolated from aged mice given three IN immunizations with 10μg RBD with PBS, NE, or NE/IVT, or IM immunizations with 10 μg RBD and Addavax two weeks after the final immunization (wk 10) and stimulated *ex vivo* with 4 μg/mL of the H-2K^b class I restricted peptide, VVLSFELL for 48h and analyzed by an IFN-γ ELISpot assay. The number of IFN-γ producing CD8 T cells are shown as spot-forming cells per 1x10^6 splenocytes as mean±SEM (n=5/grp) (**p<0.05*, ***p<0.01* by Mann-Whitney U test shown only for NE/IVT/10 RBD compared to other treatments in the same age group at the same RBD dose, and for. (**p<0.05*, ***p<0.01* by Mann-Whitney U test).

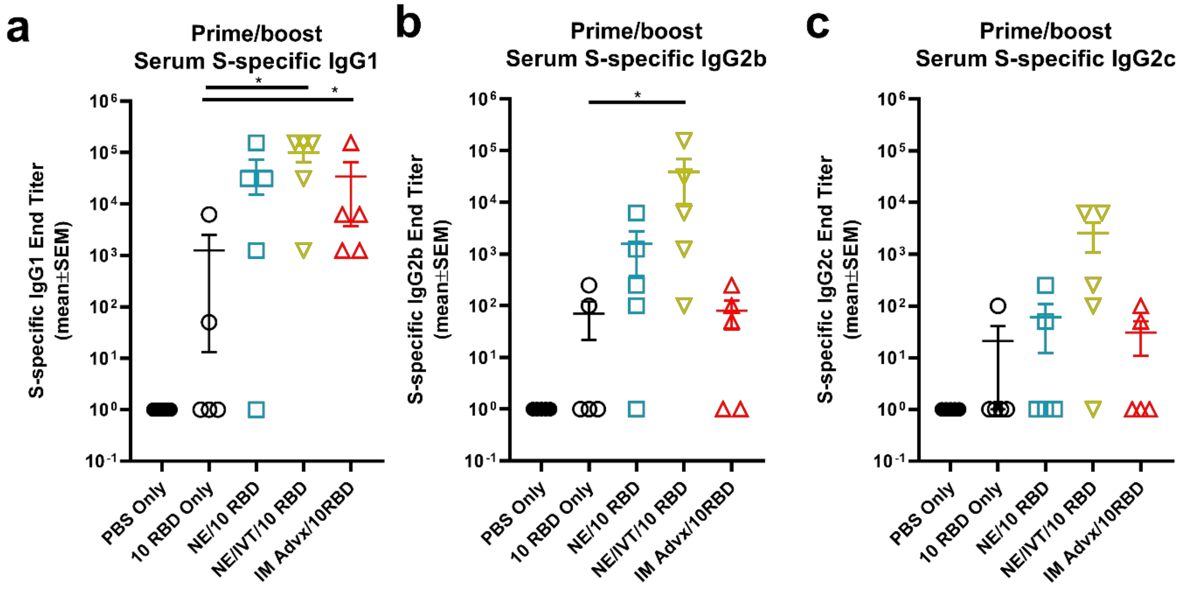

Supplementary Figure 4. Serum IgG subclass profile analysis after prime/boost immunization of senescent mice. Serum S-specific IgG subclass titers were measured two weeks after prime/boost immunization of 14-17 mo old mice described in Figure 6. (D) IgG1, (E) IgG2b, (F) IgG2c, were measured after the last immunization. Titers are shown as mean±SEM (n=5/grp) (*$p<0.05$, **$p<0.01$ by Mann-Whitney U test).

Supplementary Table 1. Complete statistical analysis of figures 1, 2, 4 and 5

TABLE S1. Complete statistical analysis of figures 1, 2, 4 and 5.

Figure 1 Total IgG, IgG subclasses, and BAL IgA (*p<0.05, **p<0.01, ***p<0.001, ns (nonsignificant) by Mann Whitney U test)

A.	IgG wk2	RBD10 old	NE/10 RBD old	NE/IVT 10 RBD old	advx/10RBD old	NE/IVT 20 RBD old	RBD10 y	NE/10 RBD y	NE/IVT/10 RBD y	advx/10RBD y	RBD20 y	NE/20 RBD y	NE/IVT/20RBD y
	RBD10 old		ns	ns	ns	ns	ns	ns	ns	ns	ns	ns	*
	NE/10 RBD old			ns	ns	ns	ns	ns	ns	ns	ns	ns	ns
	NE/IVT 10 RBD old				ns	ns	ns	ns	ns	ns	ns	ns	*
	advx/10RBD old					ns	ns	ns	ns	ns	ns	ns	*
	NE/IVT 20 RBD old						ns	ns	ns	ns	ns	ns	ns
	RBD10 y							ns	ns	ns	ns	ns	ns
	NE/10 RBD y								ns	ns	ns	ns	ns
	NE/IVT/10 RBD y									ns	ns	ns	ns
	advx/10RBD y										ns	ns	*
	RBD20 y											ns	*
	NE/20 RBD y												*
	NE/IVT/20RBD y												

B.	IgG wk6	RBD10 old	NE/10 RBD old	NE/IVT 10 RBD old	advx/10RBD old	NE/IVT 20 RBD old	RBD10 y	NE/10 RBD y	NE/IVT/10 RBD y	advx/10RBD y	RBD20 y	NE/20 RBD y	NE/IVT/20RBD y
	RBD10 old		ns	**	ns	**	ns	*	***	*	ns	**	**
	NE/10 RBD old			ns	ns	*	ns	ns	ns	ns	ns	*	*
	NE/IVT 10 RBD old				*	ns	**	ns	ns	*	**	ns	ns
	advx/10RBD old					*	ns	ns	*	ns	ns	*	*
	NE/IVT 20 RBD old						**	**	*	*	**	ns	ns
	RBD10 y							*	***	*	ns	**	**
	NE/10 RBD y								ns	ns	*	*	*
	NE/IVT/10 RBD y									**	***	ns	ns
	advx/10RBD y										*	*	*
	RBD20 y											**	**
	NE/20 RBD y												ns
	NE/IVT/20RBD y												

C.	IgG wk10	RBD10 old	NE/10 RBD old	NE/IVT 10 RBD old	advx/10RBD old	NE/IVT 20 RBD old	RBD10 y	NE/10 RBD y	NE/IVT/10 RBD y	advx/10RBD y	RBD20 y	NE/20 RBD y	NE/IVT/20RBD y
	RBD10 old		**	**	*	**	ns	***	***	**	ns	**	**
	NE/10 RBD old			ns	ns	**	**	ns	*	ns	**	**	ns
	NE/IVT 10 RBD old				*	ns	**	ns	ns	ns	**	ns	ns
	advx/10RBD old					**	**	*	**	*	**	**	*
	NE/IVT 20 RBD old						**	ns	ns	ns	**	ns	ns
	RBD10 y							***	***	**	ns	**	**
	NE/10 RBD y								ns	ns	***	ns	ns
	NE/IVT/10 RBD y									ns	***	ns	ns
	advx/10RBD y										**	ns	ns
	RBD20 y											**	**
	NE/20 RBD y												ns
	NE/IVT/20RBD y												

		A	B	C	D.	E	F	G	H	I	L	K	J
D.	IgG 1 wk10	RBD10 old	NE/10 RBD old	NE/IVT 10 RBD old	advx/10RBD old	NE/IVT 20 RBD old	RBD10 y	NE/10 RBD y	NE/IVT/10 RBD y	advx/10RBD y	RBD20 y	NE/20 RBD y	NE/IVT/20RBD y
A	RBD10 old		**	**	**	**	ns	**	**	**	ns	**	**
B	NE/10 RBD old			ns	ns	*	**	ns	ns	ns	**	*	ns
C	NE/IVT 10 RBD old				*	ns	**	ns	ns	ns	**	ns	ns
D.	advx/10RBD old					**	*	ns	*	*	**	**	*
E	NE/IVT 20 RBD old						**	*	ns	ns	**	ns	ns
F	RBD10 y							**	**	**	ns	**	**
G	NE/10 RBD y								ns	ns	**	*	ns
H	NE/IVT/10 RBD y									ns	**	ns	ns
I	advx/10RBD y										**	ns	ns
L	RBD20 y											**	**
K	NE/20 RBD y												ns
J	NE/IVT/20RBD y												

		A	B	C	D.	E	F	G	H	I	L	K	J
E.	IgG2b wk10	RBD10 old	NE/10 RBD old	NE/IVT 10 RBD old	advx/10RBD old	NE/IVT 20 RBD old	RBD10 y	NE/10 RBD y	NE/IVT/10 RBD y	advx/10RBD y	RBD20 y	NE/20 RBD y	NE/IVT/20RBD y
A	RBD10 old		**	**	ns	**	ns	**	**	**	ns	**	**
B	NE/10 RBD old			ns	ns	ns	**	ns	ns	ns	**	ns	ns
C	NE/IVT 10 RBD old				ns	ns	**	ns	ns	*	**	ns	ns
D.	advx/10RBD old					*	ns	ns	*	ns	*	*	ns
E	NE/IVT 20 RBD old						**	*	ns	*	**	ns	ns
F	RBD10 y							**	**	**	*	**	**
G	NE/10 RBD y								ns	ns	**	ns	ns
H	NE/IVT/10 RBD y									ns	**	ns	ns
I	advx/10RBD y										**	*	ns
L	RBD20 y											**	**
K	NE/20 RBD y												ns
J	NE/IVT/20RBD y												

		A	B	C	D.	E	F	G	H	I	L	K	J
F.	IgG2c wk10	RBD10 old	NE/10 RBD old	NE/IVT 10 RBD old	advx/10RBD old	NE/IVT 20 RBD old	RBD10 y	NE/10 RBD y	NE/IVT/10 RBD y	advx/10RBD y	RBD20 y	NE/20 RBD y	NE/IVT/20RBD y
A	RBD10 old		ns	ns	ns	*	ns	ns	ns	ns	ns	*	ns
B	NE/10 RBD old			ns	ns	ns	ns	ns	ns	ns	ns	ns	ns
C	NE/IVT 10 RBD old				ns	ns	ns	ns	ns	ns	ns	ns	ns
D.	advx/10RBD old					ns	ns	ns	ns	ns	ns	ns	ns
E	NE/IVT 20 RBD old						*	*	ns	ns	*	ns	ns
F	RBD10 y							ns	ns	ns	ns	*	ns
G	NE/10 RBD y								ns	ns	ns	*	ns
H	NE/IVT/10 RBD y									ns	ns	ns	ns
I	advx/10RBD y										ns	ns	ns
L	RBD20 y											ns	ns
K	NE/20 RBD y												ns
J	NE/IVT/20RBD y												

G.	IgA wk10	RBD10 old	NE/10 RBD old	NE/IVT 10 RBD old	advx/10RBD old	NE/IVT 20 RBD old	RBD10 y	NE/10 RBD y	NE/IVT/10 RBD y	advx/10RBD y	RBD20 y	NE/20 RBD y	NE/IVT/20RBD y
	RBD10 old		ns	ns	ns	ns	ns	ns	ns	ns	ns	ns	ns
	NE/10 RBD old			*	ns	**	ns	*	**	ns	ns	**	**
	NE/IVT 10 RBD old				*	ns	ns	ns	ns	ns	ns	ns	ns
	advx/10RBD old					*	ns	*	*	ns	*	*	*
	NE/IVT 20 RBD old						ns	ns	ns	ns	ns	ns	ns
	RBD10 y							ns	ns	ns	ns	ns	ns
	NE/10 RBD y								*	ns	ns	ns	ns
	NE/IVT/10 RBD y									*	**	ns	ns
	advx/10RBD y										ns	ns	ns
	RBD20 y											**	ns
	NE/20 RBD y												ns
	NE/IVT/20RBD y												

Figure 2 Neutralizing antibody titers (*p<0.05, **p<0.01, ***p<0.001, ns (nonsignificant) by Mann Whitney U test)

A.	WT MNT wk10	RBD10 old	NE/10 RBD old	NE/IVT 10 RBD old	advx/10RBD old	NE/IVT 20 RBD old	RBD10 y	NE/10 RBD y	NE/IVT/10 RBD y	advx/10RBD y	RBD20 y	NE/20 RBD y	NE/IVT/20RBD y
	RBD10 old		**	**	ns	**	ns	**	**	**	ns	**	**
	NE/10 RBD old			ns	ns	**	**	ns	*	ns	**	*	*
	NE/IVT 10 RBD old				*	**	**	ns	ns	ns	**	ns	ns
	advx/10RBD old					**	ns	**	*	ns	ns	*	*
	NE/IVT 20 RBD old						**	**	ns	**	**	*	ns
	RBD10 y							**	**	**	ns	**	**
	NE/10 RBD y								*	ns	**	*	*
	NE/IVT/10 RBD y									*	**	ns	ns
	advx/10RBD y										**	ns	*
	RBD20 y											**	**
	NE/20 RBD y												ns
	NE/IVT/20RBD y												

B.	delta MNT wk10	RBD10 old	NE/10 RBD old	NE/IVT 10 RBD old	advx/10RBD old	NE/IVT 20 RBD old	RBD10 y	NE/10 RBD y	NE/IVT/10 RBD y	advx/10RBD y	RBD20 y	NE/20 RBD y	NE/IVT/20RBD y
	RBD10 old		*	**	ns	**	ns	**	**	**	ns	**	**
	NE/10 RBD old			ns	ns	ns	*	ns	*	ns	*	ns	ns
	NE/IVT 10 RBD old				**	*	**	ns	*	ns	**	ns	ns
	advx/10RBD old					*	ns	*	**	ns	ns	**	*
	NE/IVT 20 RBD old						**	*	*	*	**	ns	ns
	RBD10 y							**	**	**	ns	**	**
	NE/10 RBD y								ns	*	**	ns	ns
	NE/IVT/10 RBD y									**	**	ns	ns
	advx/10RBD y										**	*	*
	RBD20 y											**	**
	NE/20 RBD y												ns
	NE/IVT/20RBD y												

C.	SA MNT wk10	RBD10 old	NE/10 RBD old	NE/IVT 10 RBD old	advx/10RBD old	NE/IVT 20 RBD old	RBD10 y	NE/10 RBD y	NE/IVT/10 RBD y	advx/10RBD y	RBD20 y	NE/20 RBD y	NE/IVT/20RBD y
	RBD10 old		ns	*	ns	**	ns	ns	*	**	ns	**	**
	NE/10 RBD old			ns	ns	ns	ns	ns	ns	ns	ns	ns	ns
	NE/IVT 10 RBD old				ns	ns	*	ns	ns	ns	ns	ns	ns
	advx/10RBD old					**	ns	ns	ns	ns	ns	*	**
	NE/IVT 20 RBD old						**	ns	*	*	**	*	ns
	RBD10 y							ns	*	**	ns	**	**
	NE/10 RBD y								ns	*	ns	ns	ns
	NE/IVT/10 RBD y									ns	*	ns	ns
	advx/10RBD y										*	*	*
	RBD20 y											**	**
	NE/20 RBD y												ns
	NE/IVT/20RBD y												

D.	omi MNT wk10	RBD10 old	NE/10 RBD old	NE/IVT 10 RBD old	advx/10RBD old	NE/IVT 20 RBD old	RBD10 y	NE/10 RBD y	NE/IVT/10 RBD y	advx/10RBD y	RBD20 y	NE/20 RBD y	NE/IVT/20RBD y
	RBD10 old		ns	ns	ns	**	ns	*	*	ns	ns	**	**
	NE/10 RBD old			ns	ns	*	ns	ns	ns	ns	ns	ns	*
	NE/IVT RBD old				ns	*	ns	ns	ns	ns	ns	ns	ns
	advx/10RBD old					*	ns	ns	ns	ns	ns	*	**
	NE/IVT 20 RBD old						**	ns	*	*	**	*	ns
	RBD10 y							*	ns	ns	ns	*	**
	NE/10 RBD y								ns	ns	ns	ns	ns
	NE/IVT/10 RBD y									ns	*	ns	ns
	advx/10RBD y										ns	ns	*
	RBD20 y											**	**
	NE/20 RBD y												ns
	NE/IVT/20RBD y												

Figure 4 Splenocyte T-cell recall (*p<0.05, **p<0.01, ***p<0.001, ns (nonsignificant) by Mann Whitney U test)

SPL

A.	IFNg	RBD10 old	NE/10 RBD old	NE/IVT RBD old	advx/10RBD old	NE/IVT 20 RBD old	RBD10 y	NE/10 RBD y	NE/IVT/10 RBD y	advx/10RBD y	RBD20 y	NE/20 RBD y	NE/IVT/20RBD y
	RBD10 old		ns	ns	ns	*	ns	*	ns	*	*	*	*
	NE/10 RBD old			ns	ns	**	ns	**	**	**	**	**	**
	NE/IVT 10 RBD old				ns	ns	ns	ns	ns	ns	ns	ns	ns
	advx/10RBD old					*	ns	*	ns	*	*	*	*
	NE/IVT 20 RBD old						*	ns	*	ns	ns	ns	ns
	RBD10 y							ns	ns	ns	ns	ns	*
	NE/10 RBD y								ns	ns	ns	ns	*
	NE/IVT/10 RBD y									ns	ns	ns	*
	advx/10RBD y										ns	ns	*
	RBD20 y											ns	ns
	NE/20 RBD y												ns
	NE/IVT/20RBD y												

SPL

B.	IL2	RBD10 old	NE/10 RBD old	NE/IVT 10 RBD old	advx/10RBD old	NE/IVT 20 RBD old	RBD10 y	NE/10 RBD y	NE/IVT/10 RBD y	advx/10RBD y	RBD20 y	NE/20 RBD y	NE/IVT/20RBD y
	RBD10 old		ns	ns	ns	*	ns	*	ns	ns	ns	ns	ns
	NE/10 RBD old			ns	ns	ns	ns	ns	ns	ns	ns	ns	ns
	NE/IVT 10 RBD old				*	ns	*	ns	ns	ns	ns	ns	ns
	advx/10RBD old					ns	ns	*	ns	ns	ns	ns	ns
	NE/IVT 20 RBD old						ns	ns	ns	ns	ns	ns	ns
	RBD10 y							*	ns	ns	ns	ns	ns
	NE/10 RBD y								ns	ns	ns	ns	ns
	NE/IVT/10 RBD y									ns	ns	ns	ns
	advx/10RBD y										ns	ns	ns
	RBD20 y											ns	ns
	NE/20 RBD y												ns
	NE/IVT/20RBD y												

SPL

C.	IP10	RBD10 old	NE/10 RBD old	NE/IVT 10 RBD old	advx/10RBD old	NE/IVT 20 RBD old	RBD10 y	NE/10 RBD y	NE/IVT/10 RBD y	advx/10RBD y	RBD20 y	NE/20 RBD y	NE/IVT/20RBD y
	RBD10 old		ns	ns	*	ns	ns	ns	ns	ns	*	ns	*
	NE/10 RBD old			ns	ns	*	ns	*	ns	ns	**	*	*
	NE/IVT 10 RBD old				ns	ns	ns	ns	ns	ns	ns	ns	*
	advx/10RBD old					*	ns	*	*	*	*	*	*
	NE/IVT 20 RBD old						ns	ns	ns	ns	*	ns	ns
	RBD10 y							ns	ns	ns	ns	ns	ns
	NE/10 RBD y								ns	ns	*	ns	ns
	NE/IVT/10 RBD y									ns	*	*	*
	advx/10RBD y										*	ns	*
	RBD20 y											ns	ns
	NE/20 RBD y												ns
	NE/IVT/20RBD y												

SPL

D.	TNFa	RBD10 old	NE/10 RBD old	NE/IVT 10 RBD old	advx/10RBD old	NE/IVT 20 RBD old	RBD10 y	NE/10 RBD y	NE/IVT/10 RBD y	advx/10RBD y	RBD20 y	NE/20 RBD y	NE/IVT/20RBD y
	RBD10 old		ns	ns	ns	ns	ns	ns	ns	ns	ns	ns	*
	NE/10 RBD old			ns	ns	**	ns	*	ns	*	*	**	**
	NE/IVT 10 RBD old				ns	ns	ns	ns	ns	ns	ns	ns	ns
	advx/10RBD old					*	ns	*	ns	*	*	*	*
	NE/IVT 20 RBD old						ns	ns	ns	ns	ns	ns	*
	RBD10 y							ns	ns	ns	ns	ns	*
	NE/10 RBD y								ns	ns	ns	ns	**
	NE/IVT/10 RBD y									ns	*	ns	**
	advx/10RBD y										ns	ns	**
	RBD20 y											ns	ns
	NE/20 RBD y												ns
	NE/IVT/20RBD y												

SPL

E.	IL4	RBD10 old	NE/10 RBD old	NE/IVT 10 RBD old	advx/10RBD old	NE/IVT 20 RBD old	RBD10 y	NE/10 RBD y	NE/IVT/10 RBD y	advx/10RBD y	RBD20 y	NE/20 RBD y	NE/IVT/20RBD y
	RBD10 old		ns	ns	ns	ns	ns	ns	ns	ns	**	ns	ns
	NE/10 RBD old			ns	ns	ns	ns	ns	ns	ns	ns	ns	ns
	NE/IVT 10 RBD old				ns	ns	ns	ns	ns	ns	ns	ns	ns
	advx/10RBD old					ns	ns	*	ns	*	**	ns	ns
	NE/IVT 20 RBD old						ns	ns	ns	ns	ns	ns	ns
	RBD10 y							ns	ns	ns	ns	ns	ns
	NE/10 RBD y								ns	ns	ns	ns	ns
	NE/IVT/10 RBD y									ns	ns	ns	ns
	advx/10RBD y										ns	ns	ns
	RBD20 y											ns	ns
	NE/20 RBD y												ns
	NE/IVT/20RBD y												

SPL

F.	IL5	RBD10 old	NE/10 RBD old	NE/IVT 10 RBD old	advx/10RBD old	NE/IVT 20 RBD old	RBD10 y	NE/10 RBD y	NE/IVT/10 RBD y	advx/10RBD y	RBD20 y	NE/20 RBD y	NE/IVT/20RBD y
	RBD10 old		*	ns	*	*	ns	ns	ns	*	*	ns	*
	NE/10 RBD old			ns	*	ns	*	ns	ns	*	ns	ns	ns
	NE/IVT 10 RBD old				ns	ns	ns	ns	ns	*	ns	ns	ns
	advx/10RBD old					ns	ns	*	*	ns	ns	*	*
	NE/IVT 20 RBD old						*	**	*	*	ns	*	*
	RBD10 y							ns	ns	*	ns	ns	*
	NE/10 RBD y								ns	**	ns	ns	ns
	NE/IVT/10 RBD y									**	ns	ns	ns
	advx/10RBD y										ns	**	**
	RBD20 y											ns	ns
	NE/20 RBD y												ns
	NE/IVT/20RBD y												

SPL

G.	IL13	RBD10 old	NE/10 RBD old	NE/IVT 10 RBD old	advx/10RBD old	NE/IVT 20 RBD old	RBD10 y	NE/10 RBD y	NE/IVT/10 RBD y	advx/10RBD y	RBD20 y	NE/20 RBD y	NE/IVT/20RBD y
	RBD10 old		ns	ns	*	*	ns	ns	ns	*	ns	ns	*
	NE/10 RBD old			ns	ns	*	ns	ns	ns	**	ns	ns	ns
	NE/IVT 10 RBD old				ns	ns	ns	ns	ns	ns	ns	ns	ns
	advx/10RBD old					ns	ns	*	*	ns	ns	*	ns
	NE/IVT 20 RBD old						ns	*	*	ns	ns	*	ns
	RBD10 y							ns	ns	ns	ns	ns	ns
	NE/10 RBD y								ns	ns	ns	ns	ns
	NE/IVT/10 RBD y									ns	ns	ns	ns
	advx/10RBD y										ns	ns	ns
	RBD20 y											ns	ns
	NE/20 RBD y												ns
	NE/IVT/20RBD y												

SPL													
**H.**	IL6	RBD10 old	NE/10 RBD old	NE/IVT 10 RBD old	advx/10RBD old	NE/IVT 20 RBD old	RBD10 y	NE/10 RBD y	NE/IVT/10 RBD y	advx/10RBD y	RBD20 y	NE/20 RBD y	NE/IVT/20RBD y
	RBD10 old		ns	*	*	*	ns	ns	ns	ns	ns	ns	*
	NE/10 RBD old			ns	ns	ns	ns	ns	ns	ns	ns	ns	ns
	NE/IVT 10 RBD old				ns	ns	*	**	*	ns	ns	ns	ns
	advx/10RBD old					ns	ns	*	ns	*	ns	ns	ns
	NE/IVT 20 RBD old						*	**	*	**	**	ns	ns
	RBD10 y							ns	ns	ns	*	ns	*
	NE/10 RBD y								ns	ns	*	ns	**
	NE/IVT/10 RBD y									ns	ns	ns	*
	advx/10RBD y										ns	ns	*
	RBD20 y											ns	*
	NE/20 RBD y												ns
	NE/IVT/20RBD y												

SPL		A	B	C	D	E	F	G	H	I	L	K	J
**I.**	IL17A	RBD10 old	NE/10 RBD old	NE/IVT 10 RBD old	advx/10RBD old	NE/IVT 20 RBD old	RBD10 y	NE/10 RBD y	NE/IVT/10 RBD y	advx/10RBD y	RBD20 y	NE/20 RBD y	NE/IVT/20RBD y
	RBD10 old		ns	*	ns	*	ns	ns	*	ns	*	*	*
	NE/10 RBD old			**	ns	**	ns	ns	*	ns	ns	*	**
	NE/IVT 10 RBD old				*	ns	*	**	ns	**	**	ns	ns
	advx/10RBD old					*	ns	ns	*	ns	*	*	*
	NE/IVT 20 RBD old						*	**	*	**	**	*	ns
	RBD10 y							ns	*	ns	*	*	*
	NE/10 RBD y								*	ns	ns	ns	**
	NE/IVT/10 RBD y									**	ns	ns	ns
	advx/10RBD y										**	**	**
	RBD20 y											ns	**
	NE/20 RBD y												ns
	NE/IVT/20RBD y												

SPL													
**J.**	IL10	RBD10 old	NE/10 RBD old	NE/IVT 10 RBD old	advx/10RBD old	NE/IVT 20 RBD old	RBD10 y	NE/10 RBD y	NE/IVT/10 RBD y	advx/10RBD y	RBD20 y	NE/20 RBD y	NE/IVT/20RBD y
	RBD10 old		ns	ns	ns	*	ns	ns	ns	ns	*	ns	*
	NE/10 RBD old			ns	ns	ns	ns	ns	ns	ns	ns	ns	ns
	NE/IVT 10 RBD old				ns	ns	ns	ns	ns	ns	ns	ns	ns
	advx/10RBD old					ns	ns	ns	ns	ns	ns	ns	*
	NE/IVT 20 RBD old						*	ns	ns	ns	ns	ns	ns
	RBD10 y							ns	ns	*	*	ns	*
	NE/10 RBD y								ns	ns	ns	ns	**
	NE/IVT/10 RBD y									ns	ns	ns	*
	advx/10RBD y										ns	ns	ns
	RBD20 y											ns	ns
	NE/20 RBD y												ns
	NE/IVT/20RBD y												

Figure 5 cLN T-cell recall (*p<0.05, **p<0.01, ***p<0.001, ns (nonsignificant) by Mann Whitney U test)

cLN

A.	IFNg	RBD10 old	NE/10 RBD old	NE/IVT 10 RBD old	advx/10RBD old	NE/IVT 20 RBD old	RBD10 y	NE/10 RBD y	NE/IVT/10 RBD y	advx/10RBD y	RBD20 y	NE/20 RBD y	NE/IVT/20RBD y
	RBD10 old		*	*	ns	*	ns	ns	*	ns	ns	ns	*
	NE/10 RBD old			ns	*	*	ns	ns	ns	ns	ns	ns	ns
	NE/IVT 10 RBD old				*	ns	*	*	ns	*	ns	ns	ns
	advx/10RBD old					*	ns	ns	ns	ns	ns	ns	*
	NE/IVT 20 RBD old						*	**	ns	**	*	*	ns
	RBD10 y							ns	ns	ns	ns	ns	*
	NE/10 RBD y								ns	ns	ns	ns	*
	NE/IVT/10 RBD y									ns	ns	ns	ns
	advx/10RBD y										ns	ns	*
	RBD20 y											ns	*
	NE/20 RBD y												*
	NE/IVT/20RBD y												

cLN

B.	IL2	RBD10 old	NE/10 RBD old	NE/IVT 10 RBD old	advx/10RBD old	NE/IVT 20 RBD old	RBD10 y	NE/10 RBD y	NE/IVT/10 RBD y	advx/10RBD y	RBD20 y	NE/20 RBD y	NE/IVT/20RBD y
	RBD10 old		*	*	*	*	ns	*	*	*	ns	*	*
	NE/10 RBD old			ns	ns	ns	ns	ns	ns	ns	ns	ns	ns
	NE/IVT 10 RBD old				ns	ns	ns	ns	ns	ns	*	ns	ns
	advx/10RBD old					ns	ns	ns	ns	ns	ns	ns	ns
	NE/IVT 20 RBD old						ns	ns	ns	ns	ns	ns	ns
	RBD10 y							ns	ns	ns	ns	ns	ns
	NE/10 RBD y								ns	ns	ns	ns	ns
	NE/IVT/10 RBD y									ns	*	ns	ns
	advx/10RBD y										ns	ns	ns
	RBD20 y											ns	ns
	NE/20 RBD y												ns
	NE/IVT/20RBD y												

cLN

C.	IP10	RBD10 old	NE/10 RBD old	NE/IVT 10 RBD old	advx/10RBD old	NE/IVT 20 RBD old	RBD10 y	NE/10 RBD y	NE/IVT/10 RBD y	advx/10RBD y	RBD20 y	NE/20 RBD y	NE/IVT/20RBD y
	RBD10 old		*	*	ns	*	ns	ns	*	ns	*	*	ns
	NE/10 RBD old			**	ns	*	ns	ns	*	ns	ns	ns	ns
	NE/IVT 10 RBD old				*	ns	*	**	ns	ns	ns	ns	ns
	advx/10RBD old					*	ns	ns	*	ns	ns	*	ns
	NE/IVT 20 RBD old						*	**	ns	*	ns	ns	ns
	RBD10 y							ns	*	ns	ns	*	ns
	NE/10 RBD y								*	ns	ns	*	ns
	NE/IVT/10 RBD y									ns	ns	ns	ns
	advx/10RBD y										ns	ns	ns
	RBD20 y											ns	ns
	NE/20 RBD y												ns
	NE/IVT/20RBD y												

cLN

D.	TNFa	RBD10 old	NE/10 RBD old	NE/IVT 10 RBD old	advx/10RBD old	NE/IVT 20 RBD old	RBD10 y	NE/10 RBD y	NE/IVT/10 RBD y	advx/10RBD y	RBD20 y	NE/20 RBD y	NE/IVT/20RBD y
	RBD10 old		*	*	*	*	ns	ns	*	ns	ns	ns	ns
	NE/10 RBD old			*	ns	*	ns	ns	ns	ns	ns	ns	ns
	NE/IVT 10 RBD old				ns	ns	*	**	ns	**	ns	ns	ns
	advx/10RBD old					*	ns	ns	ns	ns	ns	ns	ns
	NE/IVT 20 RBD old						*	**	ns	**	ns	ns	ns
	RBD10 y							ns	*	ns	ns	ns	ns
	NE/10 RBD y								*	ns	ns	ns	ns
	NE/IVT/10 RBD y									ns	ns	ns	ns
	advx/10RBD y										ns	ns	ns
	RBD20 y											ns	ns
	NE/20 RBD y												ns
	NE/IVT/20RBD y												

cLN

E.	IL4	RBD10 old	NE/10 RBD old	NE/IVT 10 RBD old	advx/10RBD old	NE/IVT 20 RBD old	RBD10 y	NE/10 RBD y	NE/IVT/10 RBD y	advx/10RBD y	RBD20 y	NE/20 RBD y	NE/IVT/20RBD y
	RBD10 old		ns	ns	ns	ns	ns	ns	*	ns	ns	ns	ns
	NE/10 RBD old			ns	ns	ns	ns	ns	**	ns	ns	ns	ns
	NE/IVT 10 RBD old				ns	ns	ns	ns	ns	ns	ns	ns	ns
	advx/10RBD old					ns	ns	ns	*	ns	ns	ns	ns
	NE/IVT 20 RBD old						ns	ns	ns	ns	ns	ns	ns
	RBD10 y							ns	*	ns	ns	ns	ns
	NE/10 RBD y								*	ns	ns	ns	ns
	NE/IVT/10 RBD y									ns	**	ns	ns
	advx/10RBD y										ns	ns	ns
	RBD20 y											ns	ns
	NE/20 RBD y												ns
	NE/IVT/20RBD y												

cLN

F.	IL5	RBD10 old	NE/10 RBD old	NE/IVT 10 RBD old	advx/10RBD old	NE/IVT 20 RBD old	RBD10 y	NE/10 RBD y	NE/IVT/10 RBD y	advx/10RBD y	RBD20 y	NE/20 RBD y	NE/IVT/20RBD y
	RBD10 old		*	*	*	*	ns	*	*	*	*	*	*
	NE/10 RBD old			**	*	**	ns	ns	**	**	ns	ns	**
	NE/IVT 10 RBD old				ns	ns	*	ns	ns	*	*	ns	ns
	advx/10RBD old					ns	ns	ns	ns	*	*	ns	ns
	NE/IVT 20 RBD old						*	ns	ns	*	**	ns	ns
	RBD10 y							ns	*	*	ns	ns	*
	NE/10 RBD y								ns	*	ns	ns	ns
	NE/IVT/10 RBD y									*	**	ns	ns
	advx/10RBD y										**	**	*
	RBD20 y											ns	*
	NE/20 RBD y												ns
	NE/IVT/20RBD y												

cLN

G.	IL13	RBD10 old	NE/10 RBD old	NE/IVT 10 RBD old	advx/10RBD old	NE/IVT 20 RBD old	RBD10 y	NE/10 RBD y	NE/IVT/10 RBD y	advx/10RBD y	RBD20 y	NE/20 RBD y	NE/IVT/20RBD y
	RBD10 old		ns	ns	*	*	ns	ns	*	*	ns	*	*
	NE/10 RBD old			ns	*	**	ns	ns	ns	**	ns	ns	ns
	NE/IVT 10 RBD old				ns	ns	ns	ns	ns	ns	ns	ns	ns
	advx/10RBD old					ns	ns	ns	ns	*	ns	ns	ns
	NE/IVT 20 RBD old						*	*	ns	ns	*	ns	ns
	RBD10 y							ns	ns	*	ns	ns	ns
	NE/10 RBD y								ns	*	ns	ns	ns
	NE/IVT/10 RBD y									ns	ns	ns	ns
	advx/10RBD y										**	*	*
	RBD20 y											ns	ns
	NE/20 RBD y												ns
	NE/IVT/20RBD y												

cLN													
**H.**	IL6	RBD10 old	NE/10 RBD old	NE/IVT 10 RBD old	advx/10RBD old	NE/IVT 20 RBD old	RBD10 y	NE/10 RBD y	NE/IVT/10 RBD y	advx/10RBD y	RBD20 y	NE/20 RBD y	NE/IVT/20RBD y
	RBD10 old		*	*	*	*	ns	ns	*	*	ns	*	*
	NE/10 RBD old			*	ns	*	*	ns	ns	ns	ns	*	*
	NE/IVT 10 RBD old				ns	ns	*	**	ns	ns	ns	ns	ns
	advx/10RBD old					*	ns	ns	ns	ns	ns	ns	ns
	NE/IVT 20 RBD old						*	**	*	*	ns	ns	ns
	RBD10 y							ns	*	*	ns	*	*
	NE/10 RBD y								ns	ns	ns	**	*
	NE/IVT/10 RBD y									ns	ns	ns	ns
	advx/10RBD y										ns	ns	ns
	RBD20 y											ns	ns
	NE/20 RBD y												ns
	NE/IVT/20RBD y												

cLN													
**I.**	IL17A	RBD10 old	NE/10 RBD old	NE/IVT 10 RBD old	advx/10RBD old	NE/IVT 20 RBD old	RBD10 y	NE/10 RBD y	NE/IVT/10 RBD y	advx/10RBD y	RBD20 y	NE/20 RBD y	NE/IVT/20RBD y
	RBD10 old		*	*	ns	*	ns	*	*	ns	ns	*	*
	NE/10 RBD old			**	*	**	*	ns	*	**	ns	*	**
	NE/IVT 10 RBD old				*	ns	*	**	ns	**	**	ns	ns
	advx/10RBD old					*	ns	ns	*	ns	ns	*	*
	NE/IVT 20 RBD old						*	**	ns	**	**	ns	ns
	RBD10 y							*	*	ns	ns	ns	*
	NE/10 RBD y								*	*	ns	*	**
	NE/IVT/10 RBD y									**	*	ns	ns
	advx/10RBD y										ns	**	**
	RBD20 y											**	**
	NE/20 RBD y												ns
	NE/IVT/20RBD y												

cLN													
**J.**	IL10	RBD10 old	NE/10 RBD old	NE/IVT 10 RBD old	advx/10RBD old	NE/IVT 20 RBD old	RBD10 y	NE/10 RBD y	NE/IVT/10 RBD y	advx/10RBD y	RBD20 y	NE/20 RBD y	NE/IVT/20RBD y
	RBD10 old		ns	ns	ns	*	ns	ns	*	*	ns	ns	*
	NE/10 RBD old			ns	ns	**	ns	ns	ns	ns	ns	ns	ns
	NE/IVT 10 RBD old				ns	ns	*	*	ns	ns	*	ns	ns
	advx/10RBD old					*	ns	*	ns	ns	ns	ns	ns
	NE/IVT 20 RBD old						*	**	ns	ns	**	ns	ns
	RBD10 y							ns	*	ns	ns	ns	ns
	NE/10 RBD y								*	*	ns	ns	*
	NE/IVT/10 RBD y									ns	*	ns	ns
	advx/10RBD y										ns	ns	ns
	RBD20 y											ns	*
	NE/20 RBD y												ns
	NE/IVT/20RBD y												
