# Supplementary material for: Multicomponent intranasal adjuvant for mucosal and durable systemic SARS-CoV-2 immunity in young and aged mice
Source: NPJ Vaccines. 2023 Jun 29;8:96. doi: 10.1038/s41541-023-00691-1 (PMC10310740; doi:10.1038/s41541-023-00691-1)
Supplement: Supplementary file 2 — REPORTING SUMMARY [file 41541_2023_691_MOESM2_ESM.pdf]

nature research

Corresponding author(s): Pamela T. Wong, Michael Schotsaert

# Reporting Summary

Nature Research wishes to improve the reproducibility of the work that we publish. This form provides structure for consistency and transparency in reporting. For further information on Nature Research policies, see Authors & Referees and the Editorial Policy Checklist.

## Statistical parameters

When statistical analyses are reported, confirm that the following items are present in the relevant location (e.g. figure legend, table legend, main text, or Methods section).

| n/a | Confirmed | |
|---|---|---|
| ☐ | ☒ | The exact sample size (*n*) for each experimental group/condition, given as a discrete number and unit of measurement |
| ☐ | ☒ | An indication of whether measurements were taken from distinct samples or whether the same sample was measured repeatedly |
| ☐ | ☒ | The statistical test(s) used AND whether they are one- or two-sided<br>*Only common tests should be described solely by name; describe more complex techniques in the Methods section.* |
| ☐ | ☒ | A description of all covariates tested |
| ☐ | ☒ | A description of any assumptions or corrections, such as tests of normality and adjustment for multiple comparisons |
| ☐ | ☒ | A full description of the statistics including central tendency (e.g. means) or other basic estimates (e.g. regression coefficient) AND variation (e.g. standard deviation) or associated estimates of uncertainty (e.g. confidence intervals) |
| ☒ | ☐ | For null hypothesis testing, the test statistic (e.g. *F*, *t*, *r*) with confidence intervals, effect sizes, degrees of freedom and *P* value noted<br>*Give P values as exact values whenever suitable.* |
| ☒ | ☐ | For Bayesian analysis, information on the choice of priors and Markov chain Monte Carlo settings |
| ☒ | ☐ | For hierarchical and complex designs, identification of the appropriate level for tests and full reporting of outcomes |
| ☒ | ☐ | Estimates of effect sizes (e.g. Cohen's *d*, Pearson's *r*), indicating how they were calculated |
| ☐ | ☒ | Clearly defined error bars<br>*State explicitly what error bars represent (e.g. SD, SE, CI)* |

*Our web collection on statistics for biologists may be useful.*

## Software and code

Policy information about availability of computer code

| | |
|---|---|
| Data collection | No software was used |
| Data analysis | Graphpad Prism |

For manuscripts utilizing custom algorithms or software that are central to the research but not yet described in published literature, software must be made available to editors/reviewers upon request. We strongly encourage code deposition in a community repository (e.g. GitHub). See the Nature Research guidelines for submitting code & software for further information.

## Data

Policy information about availability of data

All manuscripts must include a data availability statement. This statement should provide the following information, where applicable:

- Accession codes, unique identifiers, or web links for publicly available datasets
- A list of figures that have associated raw data
- A description of any restrictions on data availability

All data generated or analyzed for these studies are included in this published article (and its supplementary information files) and/or are available upon request from the corresponding authors.



March 2018

# Field-specific reporting

Please select the best fit for your research. If you are not sure, read the appropriate sections before making your selection.

☒ Life sciences ☐ Behavioural & social sciences

For a reference copy of the document with all sections, see nature.com/authors/policies/ReportingSummary-flat.pdf

# Life sciences

## Study design

All studies must disclose on these points even when the disclosure is negative.

| | |
|---|---|
| Sample size | The number of mice used for each study was calculated based on power analyses and the results of previous studies of short and long term evaluation of the immune response and what was necessary for achieving statistical relevance as assessed by Mann-Whitney U tests. |
| Data exclusions | no data was excluded |
| Replication | The data presented have been replicated at least once and also replicated in other mouse strains as well (Balb/c young and aged). |
| Randomization | These studies used inbred mouse strains shipped at the same time at the same age of life, and thus randomization was not required. The challenge study in senescent mice however, used mice ranging from 14-17 months of age which were distributed equally into the different treatment groups. |
| Blinding | Blinding was not critical to these studies as readouts were all quantitative values-absorbance/luminescence (no interpretation needed as in pathology scoring for example). However, technicians and students were only given group numbers (rather than treatment type) at the time of sample collection. |

## Materials & experimental systems

Policy information about availability of materials

| n/a | Involved in the study |
|---|---|
| ☐ | ☒ Unique materials |
| ☐ | ☒ Antibodies |
| ☐ | ☒ Eukaryotic cell lines |
| ☐ | ☒ Research animals |
| ☒ | ☐ Human research participants |

### Unique materials

| | |
|---|---|
| Obtaining unique materials | Unique materials are available upon request from the authors for research purposes. |

### Antibodies

| | |
|---|---|
| Antibodies used | Antibodies used included secondary antibodies for detection conjugated to alkaline phosphatase: goat-anti-mouse IgG (H+L) (Jackson ImmunoResearch Laboratories cat. 115-055-003); goat-anti-mouse IgG1 (Jackson ImmunoResearch Laboratories cat. 115-055-205); goat-anti-mouse IgG2b (Jackson ImmunoResearch Laboratories cat.115-055-207); goat-anti-mouse IgG2c (Jackson ImmunoResearch Laboratories cat.115-055-208); rabbit-anti-mouse IgA (Rockland cat. 610-4506). Antibodies used for plaque detection included: mouse anti-SARS-CoV-1/2 nucleoprotein (clone 1C7C7) and mouse anti-SARS-CoV-2 spike (clone 2B3E5) monoclonal antibodies (produced by the Center for Therapeutic Antibody Development at the Icahn School of Medicine at Mount Sinai), and HRP-conjugated goat-anti-mouse IgG (Abcam clone 2B3E5 cat ab6823). |
| Validation | These antibodies have been verified for specificity and also species cross reactivity. |

### Eukaryotic cell lines

Policy information about cell lines

| | |
|---|---|
| Cell line source(s) | HEK293T-hACE2 cells were obtained from BEI Resources, Vero E6 cells were obtained from ATCC. |
| Authentication | These lines were authenticated with certificates of analysis. |
| Mycoplasma contamination | Cell lines were validated for lack of mycoplasma contamination at BEI/ATCC. |

Commonly misidentified lines
(See ICLAC register)

*Name any commonly misidentified cell lines used in the study and provide a rationale for their use.*

## Research animals

Policy information about studies involving animals; ARRIVE guidelines recommended for reporting animal research

Animals/animal-derived materials

C57Bl/6 female mice were used for all immunization studies. For young mice 6-8 wk old mice were used, for aged mice, 8 month old mice were used, and for senescent mice, 14-17 month old mice were used.

# Method-specific reporting

| n/a | Involved in the study |
| --- | --- |
| ☒ | ☐ ChIP-seq |
| ☒ | ☐ Flow cytometry |
| ☒ | ☐ Magnetic resonance imaging |
